# Signature White Matter Hyperintensity Locations Associated With Vascular Risk Factors Derived From 15 653 Individuals

**DOI:** 10.1161/STROKEAHA.125.051159

**Published:** 2025-08-15

**Authors:** J. Matthijs Biesbroek, Floor A.S. de Kort, Devasuda Anblagan, Mark E. Bastin, Alexa Beiser, Henry Brodaty, Nishi Chaturvedi, Christopher P.L.H. Chen, Bastian Cheng, Ching-Yu Cheng, Simon R. Cox, Charles DeCarli, Christian Enzinger, Evan Fletcher, Richard Frayne, Marius de Groot, Saima Hilal, Felicia Huang, M. Arfan Ikram, Jiyang Jiang, Bonnie Y.K. Lam, Pauline Maillard, Carola Mayer, Cheryl R. McCreary, Vincent Mok, Susana Muñoz Maniega, Marvin Petersen, Genady Roshchupkin, Perminder S. Sachdev, Reinhold Schmidt, Stephan Seiler, Sudha Seshadri, Carole H. Sudre, Götz Thomalla, Maria Valdés Hernández, Narayanaswamy Venketasubramanian, Meike W. Vernooij, Elisabeth J. Vinke, Joanna M. Wardlaw, Wei Wen, Hugo J. Kuijf, Geert Jan Biessels

**Affiliations:** Department of Neurology, University Medical Center Utrecht Brain Center, the Netherlands (J.M.B., F.A.S.d.K., G.J.B.).; Department of Neurology, Diakonessenhuis Hospital, Utrecht, the Netherlands (J.M.B.).; Centre for Clinical Brain Sciences (D.A., M.E.B., S.M.M., M.V.H., J.M.W.), The University of Edinburgh, United Kingdom.; Lothian Birth Cohorts, Department of Psychology (S.R.C.), The University of Edinburgh, United Kingdom.; UK Dementia Research Institute (J.M.W.), The University of Edinburgh, United Kingdom.; Department of Biostatistics, Boston University School of Public Health, MA (A.B.).; Centre for Healthy Brain Ageing, Discipline of Psychiatry and Mental Health, School of Clinical Medicine, University of New South Wales, Sydney, Australia (H.B., J.J., P.S.S., W.W.).; Unit for Lifelong Health and Ageing, Department of Population Science and Experimental Medicine (N.C., F.H., C.H.S.), University College London, United Kingdom.; Department of Computer Science, Hawkes Institute (C.H.S.), University College London, United Kingdom.; Department of Pharmacology, Yong Loo Lin School of Medicine, National University of Singapore (C.P.L.H.C., S.H.).; Memory, Aging and Cognition Center, National University Health System, Singapore (C.P.L.H.C., S.H., N.V.).; Department of Neurology, University Medical Hospital Hamburg-Eppendorf, Germany (B.C., C.M., M.P., G.T.).; Singapore Eye Research Institute, Singapore National Eye Center (C.-Y.C.).; Department of Neurology, University of California Davis, Sacramento (C.D., E.F., P.M.).; Department of Neurology, Medical University Graz, Austria (C.E., R.S., S. Seiler).; Department of Clinical Neurosciences (R.F., C.R.M.), Hotchkiss Brain Institute, University of Calgary, AB, Canada.; Department of Radiology (R.F., C.R.M.), Hotchkiss Brain Institute, University of Calgary, AB, Canada.; Centre for Neuroimaging Sciences, Institute of Psychiatry, Psychology and Neuroscience (M.d.G.), King’s College London, United Kingdom.; Department of Biomedical Computing, School of Biomedical Engineering and Imaging Sciences (C.H.S.), King’s College London, United Kingdom.; Saw Swee Hock School of Public Health, National University of Singapore and National University Health System (S.H.).; Department of Epidemiology (M.A.I., G.R., M.W.V., E.J.V.), Erasmus MC University Medical Center, Rotterdam, the Netherlands.; Department of Radiology and Nuclear Medicine (G.R., M.W.V., E.J.V.), Erasmus MC University Medical Center, Rotterdam, the Netherlands.; Faculty of Medicine, Division of Neurology, Department of Medicine and Therapeutics, The Chinese University of Hong Kong (B.Y.K.L., V.M.).; Glenn Biggs Institute for Alzheimer’s and Neurodegenerative Diseases, University of Texas Health Science Center, San Antonio (S. Seshadri).; Raffles Neuroscience Center, Raffles Hospital, Singapore (N.V.).; Faculty of Applied Sciences, Delft University of Technology, the Netherlands (M.W.V.).; Image Sciences Institute, University Medical Center Utrecht, the Netherlands (H.J.K.).

**Keywords:** arteriolosclerosis, cerebral amyloid angiopathy, cerebral small vessel diseases, hypertension, vascular diseases

## Abstract

**BACKGROUND:**

White matter hyperintensities (WMHs) of presumed vascular origin are common in the elderly and are associated with vascular risk factors. There is evidence that vascular risk factors, in particular hypertension, are associated with WMH in particular locations of the white matter. However, it remains unclear whether this is true for all risk factors and whether signature WMH locations differ between risk factors. We aimed to identify WMH locations associated with vascular risk factors in community-dwelling individuals.

**METHODS:**

We pooled cross-sectional data from 16 population-based cohorts (15 653 individuals; mean age, 64.2±11.8 years; 52.2% female) through the Meta VCI Map Consortium. We quantified associations between WMH volumes in 50 white matter regions and 6 vascular risk factors using linear mixed models. Analyses were corrected for age, sex, study site, and total WMH volume.

**RESULTS:**

Hypertension (B=0.141; *P*<0.001), smoking (B=0.096; *P*<0.001), diabetes (B=0.059; *P*<0.001), and history of vascular disease (B=0.056; *P*=0.034) were significantly associated with higher total WMH volume, whereas obesity (B=0.023; *P*=0.139) and hypercholesterolemia (B=0.009; *P*=0.531) were not. After correcting for total WMH volume, hypertension was associated with WMH volume in 10 regions (ie, bilateral external capsule, superior longitudinal fasciculus, superior corona radiata, anterior limb of the internal capsule, left anterior corona radiata, and left superior fronto-occipital fasciculus), smoking (body corpus callosum), diabetes (genu corpus callosum), and obesity (left inferior fronto-occipital fasciculus), each with one region.

**CONCLUSIONS:**

Hypertension has a signature WMH pattern, whereas associations between other vascular risk factors and regional WMH volumes seem to be mainly explained by a global increase in WMH rather than region-specific effects.

White matter hyperintensities (WMHs) of presumed vascular origin are common in the elderly.^1,2^ WMHs are a heterogeneous entity and may reflect several causes and disease mechanisms. The commonest cause of WMH is cerebral small vessel diseases in the context of arteriolosclerosis or cerebral amyloid angiopathy pathologies.^3,4^ However, WMH may also be caused by neurodegenerative, metabolic, or inflammatory disease processes.^3,5,6^ Identifying underlying disease mechanisms of WMH and understanding how these mechanisms affect the white matter are a priority research topic and may enable novel and individualized treatment strategies.^4^ There is increasing evidence that specific disease mechanisms may have signature WMH patterns and locations.^7^ Such patterns might provide clues on underlying disease mechanisms.

Most evidence for an association between WMH location and underlying disease mechanisms comes from studies that have focused on vascular risk factors or amyloid pathology.^7–9,13,15–21^ The overall findings from these prior studies suggest that amyloid pathology is primarily associated with posterior WMH and vascular risk factors with more anterior WMH (Table 1).

**Table 1.**
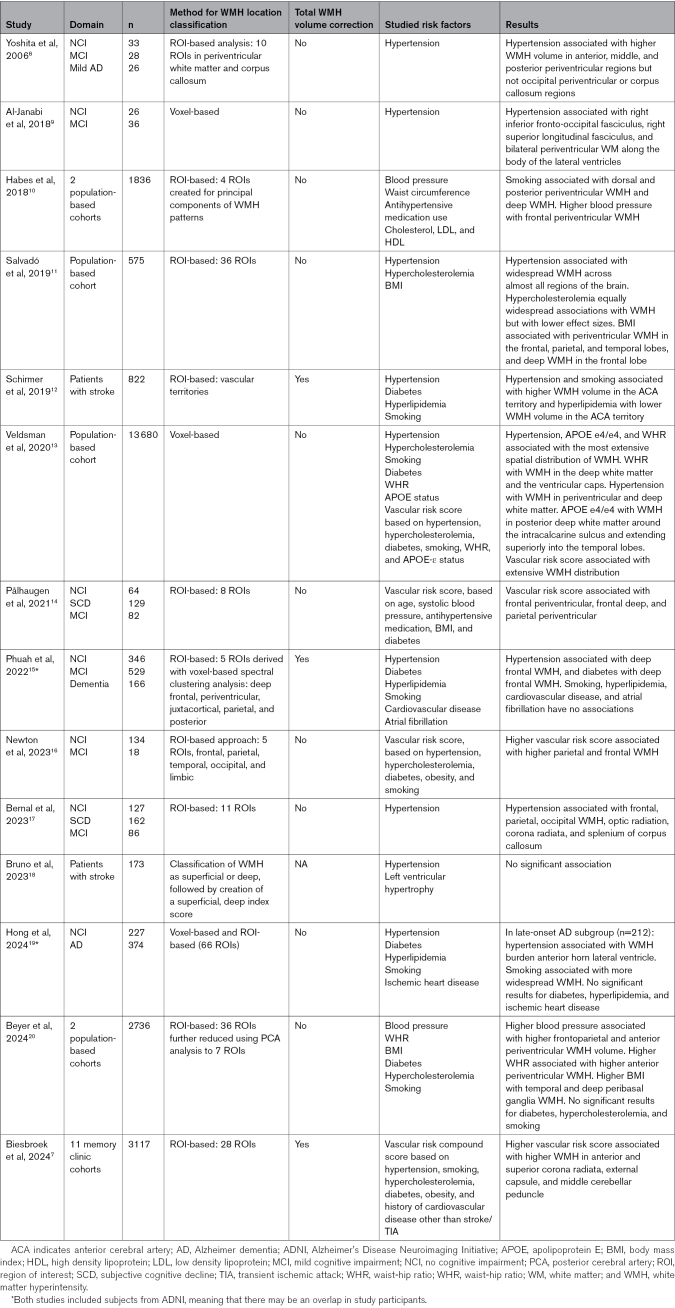
Summary of Prior Studies on the Relation Between WMH Location and Vascular Risk Factors

However, several important limitations should be noted. First, nearly all these studies used different methods for parcellation of the white matter and WMH location classification, which hampers a detailed comparison of the results. In addition, when focusing on vascular risk factors, many studies focused only on hypertension^8,9,17,18^ or a vascular risk compound score (VRCS) with varying definitions.^7,16^ There are limited data on WMH locations associated with other individual vascular risk factors. Thus, the association between vascular risk factors and anterior WMH location might be primarily driven by ≥1 specific risk factors (in which case, hypertension seems a likely candidate), and the question remains whether different risk factors have different signature WMH locations. Finally, most studies did not correct for total WMH volume, meaning that associations between vascular risk factors and regional WMH volumes may (in part) be explained by a global increase in WMH and may not reflect region-specific effects.

In this study, we aimed to identify and compare WMH location associated with 6 individual vascular risk factors in a large multicenter study comprising 15 653 individuals from 16 population-based cohorts.

## Methods

The data that support the findings of this study are available from the corresponding author/project leads upon reasonable request. Restrictions related to privacy and personal data sharing regulations and informed consent may apply. This article follows the Strengthening the Reporting of Observational Studies in Epidemiology statement guidelines for reporting observational studies.

### Participant Selection

We harmonized individual-level data from 16 cohorts across 9 countries spanning 4 continents: Australia (MAS [Sydney Memory and Ageing Study] and OATS [Older Australian Twin Study]), Austria (ASPS [Austrian Stroke Prevention Study] and ASPSF [Austrian Stroke Prevention Study Family]), Canada (CNS [Calgary Normative Study]), China (CU-RISK [Chinese University of Hong Kong Risk Index for Subclinical Brain Lesions in Hong Kong Study), Germany (HCHS [Hamburg City Health Study]), the Netherlands (RS [Rotterdam Study]), Singapore (EDIS [Epidemiology of Dementia in Singapore Study]^22^), the United Kingdom (SABRE [Southall and Brent Revisited Study], LBC1921 [Lothian Birth Cohort 1921], and LBC1936 [Lothian Birth Cohort 1936]), and the United States (FHS_Gen2 [Framingham Heart Study_Gen2], FHS_Gen3 [Framingham Heart Study_Gen3], FHS_Omni1 [Framingham Heart Study_Omni1], and AUCD [Alzheimer's Disease UC Davis Diversity Cohort]) through the Meta VCI Map Consortium (www.metavcimap.org). Background and organization of the Meta VCI Map Consortium are described in the design article^23^ and on the consortium website (http://www.metavcimap.org). Cohorts were eligible for inclusion if participants were recruited from the general population and underwent brain magnetic resonance imaging (MRI; with availability of fluid attenuated inversion recovery and T1 sequences). Data from 15 cohorts (all cohorts except EDIS) were available from a prior consortium project, the details of which are described elsewhere.^2^ Data from the EDIS cohort were available from a prior lesion-symptom mapping study.^22^ Cohort details including study design, recruitment period, inclusion criteria, and exclusion criteria are provided in Table S1. Individuals with failed brain MRI processing (n=22), missing clinical data on age, sex, or outcome of evaluation for dementia (n=26), a diagnosis of dementia (n=50), and subjects with missing data for all 6 vascular risk factors (n=13) were excluded. Individuals with mild cognitive impairment or a history of stroke were eligible for inclusion. The flowchart of the final participant selection is shown in Figure S1. For all cohorts, ethical and institutional approval was obtained as required by local regulations to allow data acquisition, including informed consent and data sharing.

### Vascular Risk Factors

Vascular risk factors considered included hypertension, current smoking, hypercholesterolemia, diabetes, obesity, and history of a vascular event other than stroke or transient ischemic attack. Definitions and harmonization procedures for each of the vascular risk factors are provided in the Supplemental Material. A VRCS was calculated following a previously described method^7,24^ by summing up the 6 aforementioned factors, giving equal weight to each risk factor (thus, the VRCS ranged from 0 to 6). The VRCS was subsequently expressed as a proportion, ranging from 0 to 1 (ie, the number of present factors was divided by the number of available factors for each individual). To account for missing variables, the VRCS was only calculated if data on a minimum of 3 risk factors were available.

### Brain MRI Processing

MRI protocols and details of the procedures for WMH segmentation and registration are provided in the previously published Meta VCI Map Consortium^2^ and EDIS projects^22^ from which the WMH maps were reused. In short, binary WMH segmentations were provided by the participating centers or automatically computed in Utrecht. WMH segmentations were registered to the 1 mm × 1 mm× 1 mm resolution Montreal Neurological Institute-152 brain template^25^ for spatial normalization. Voxels located outside the white matter (defined using the Montreal Neurological Institute probabilistic white matter atlas, thresholded at 30% white matter probability) were removed from all individual WMH segmentations to minimize the effect of possible misclassifications of other lesion types as WMH. The ICBM-DTI-81 (International Consortium of Brain Mapping Diffusion Tensor Imaging 81) white matter atlas^26^ in Montreal Neurological Institute-152 space was used to calculate WMH volumes in 50 regions of interest (ROIs). Volumes were calculated in milliliters and cube-root transformed to obtain a normal data distribution and standardized (ie, transformed to *Z* scores) before performing regression analyses. All analyses were performed in Montreal Neurological Institute-152 space and are, thus, corrected (ie, normalized) for differences in intracranial volume.

### Statistical Analysis

Individual vascular risk factors were analyzed as dichotomous variables. The VRCS was transformed into a standardized *Z* score and analyzed as a continuous variable. A complete case analysis was conducted separately for each vascular risk factor. As a result, the number of included participants varied across models. Linear mixed models were used to determine the relation between each of the vascular risk factors (in separate models) or the VRCS as determinant and total and regional WMH volumes as dependent variables. All analyses were corrected for age and sex (as fixed effects) and study site (as a random effect). Analyses with regional WMH volumes as the dependent variable were performed before and after additional correction for total WMH volume. To account for multiple comparisons (ie, for analyzing 50 ROIs), a Bonferroni correction was applied, and thus, an uncorrected *P* value of <0.001 was considered statistically significant. Associations between risk factors and total WMH volume, as well as any statistically significant association between a specific vascular risk factor and regional WMH volumes after correction for age, sex, study site, and total WMH volume, were subsequently additionally corrected for the presence of all other vascular risk factors in a subsample of participants for whom data on all 6 risk factors were available.

Post hoc analyses were performed to explore possible age effects on the relation between vascular risk factors and WMH volumes. For all statistically significant associations between either a vascular risk factor and total WMH volume or between a vascular risk factor and regional WMH volume after correction for total WMH volume, an interaction term for the vascular risk factor with age was added to the model. If the interaction term was statistically significant, models were subsequently stratified for age, using 3 age strata: young (<60 years), old (60–80 years), and very old (>80 years) participants, before and after restandardizing WMH volumes within age strata. Finally, a sensitivity analysis of the main results was performed including only subjects without a history of stroke.

All reported coefficients (B) can be interpreted as standardized coefficients because the independent (VRCS) and dependent variables (WMH volumes) were transformed to *Z* scores before analysis. The coefficients reported for individual vascular risk factors are the difference in *Z* scores of cube-root transformed WMH volumes for individuals with versus individuals without the risk factor.

## Results

### Participant Characteristics

We analyzed 15 653 individuals covering the ages of 18 to 97 years. The merged cohort included 16 population cohorts from North America, Europe, Asia, and Oceania. Despite the large geographic coverage, 83.8% (n=13 105) of the cohort was of White race and ethnicity. Mean age was 64.2 (SD, 11.8) years; 52.2% (n=8170) of the participants were female. The number of individuals with available data for each risk factor was 15 633 for hypertension, 15 532 for current smoking, 15 407 for diabetes, 14 946 for hypercholesterolemia, 14 637 for obesity, and 11 106 for history of cardiovascular disease. Hypertension (58.3%) and hypercholesterolemia (40.0%) were the most prevalent risk factors, followed by obesity (21.2%), diabetes (17.1%), current smoking (11.8%), and history of cardiovascular disease other than stroke or transient ischemic attack (9.1%). The mean VRCS was 0.28 (SD, 0.22 [range, 0–1]). A WMH prevalence map is provided in Figure S2. Further details about participant characteristics are provided in Table 2.

**Table 2.**
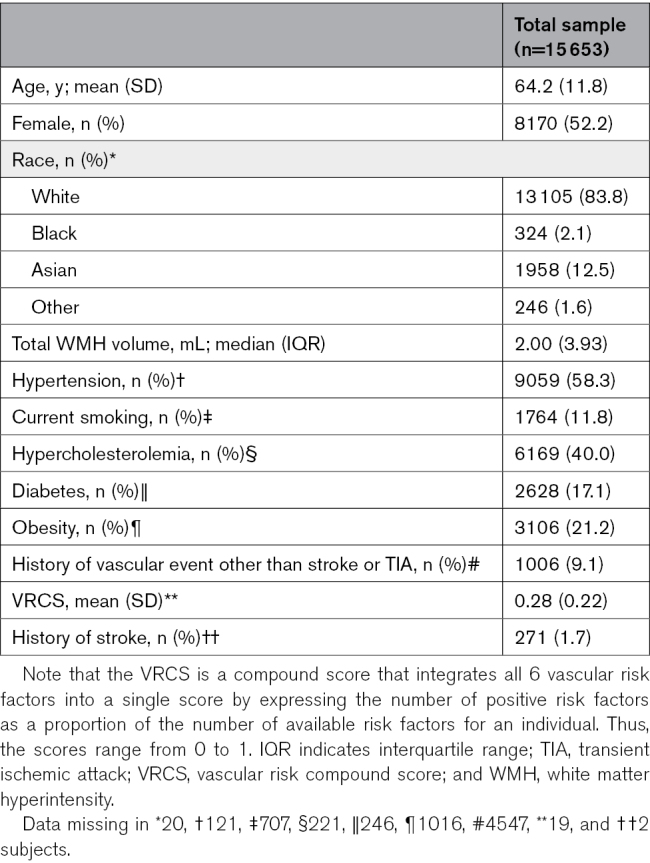
Participant Characteristics

### Associations Between Vascular Risk Factors and Total WMH Volume

After correcting for age, sex, and study site, presence of hypertension (B=0.141; *P*<0.001) had the largest coefficient in relation to total WMH volume, followed by current smoking (B=0.096; *P*<0.001), diabetes (B=0.059; *P*<0.001), and history of cardiovascular disease (B=0.056; *P*=0.034). Obesity (B=0.023; *P*=0.139) and hypercholesterolemia (B=0.009; *P*=0.531) were not associated with total WMH volume. A higher VRCS was associated with higher total WMH volume (B=0.056; *P*<0.001). In the multivariable model that included all 6 vascular risk factors, hypertension (B=0.123; *P*<0.001), current smoking (B=0.096; *P*<0.001), and diabetes (B=0.071; *P*=0.003) were each independently associated with total WMH volume, whereas history of cardiovascular disease, obesity, and hypercholesterolemia were not. Further details are provided in Table 3.

**Table 3.**
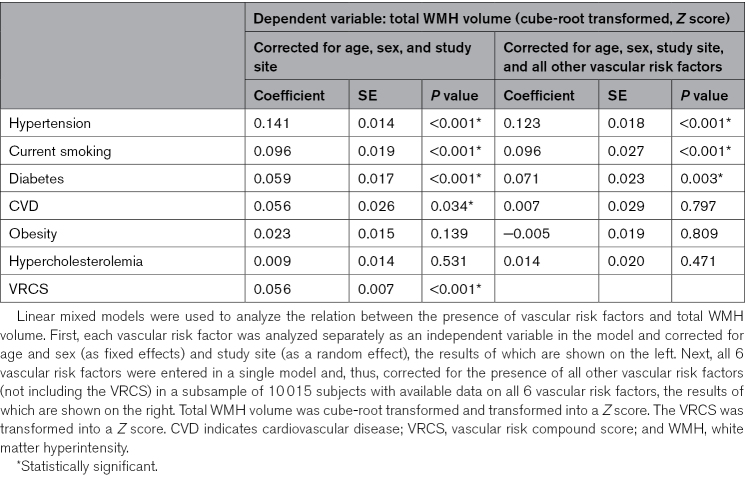
Associations Between Vascular Risk Factors and Total WMH Volume

#### Post Hoc Age-Stratified Analyses

Hypertension (*P*<0.001) and smoking (*P*=0.035), but not diabetes, significantly interacted with age in relation to total WMH volume. In stratified analyses, overall association magnitudes were highest in individuals aged 60 to 80 years (hypertension: n=9796; B=0.17; *P*<0.001; smoking: n=9279; B=0.15; *P*<0.001) and lower in individuals aged <60 years (hypertension: n=4888; B=0.08; *P*<0.001; smoking: n=4643; B=0.05; *P*=0.002), and results were not significant in individuals aged >80 years (hypertension: n=848; B=0.00; *P*=0.982; smoking: n=715; B=0.08; *P*=0.618). After restandardizing *Z* scores of WMH volumes within each age stratum, coefficients in individuals aged <60 years (hypertension: B=0.13; smoking: B=0.09) and individuals aged 60 to 80 years (hypertension: B=0.18; smoking: B=0.15) were more similar, and coefficients remained lowest in individuals aged >80 years (hypertension: B=0.00; smoking: B=0.06).

### Associations Between Vascular Risk Factors and Regional WMH Volumes

A detailed overview of the coefficients, standard errors, and *P* values of all associations between vascular risk factors and regional WMH volumes is provided in Tables 4 and 5 and Tables S2 through S8. After correction for age, sex, and study site, hypertension was significantly associated with regional WMH volume in 28 of 50 tested regions, with largely symmetrical patterns. Coefficients were highest for the bilateral external capsule (B=0.18), superior longitudinal fasciculus (B=0.17), and superior and corona radiata (B=0.16). After additional correction for total WMH volume, associations between hypertension and regional WMH volumes remained significant for 10 regions, namely, the bilateral external capsule, superior longitudinal fasciculus, superior corona radiata, and anterior limb of the internal capsule, as well as the left anterior corona radiata and left superior fronto-occipital fasciculus (Table 4). A visual representation of the locations of the significant regions for hypertension is provided in the Figure. After additional correction for the presence of other vascular risk factors (in a subset of 10 015 individuals who had available data on all 6 risk factors), the coefficients of the associations between hypertension and WMH volume in these 10 regions remained essentially unchanged (Table 5).

**Table 4.**
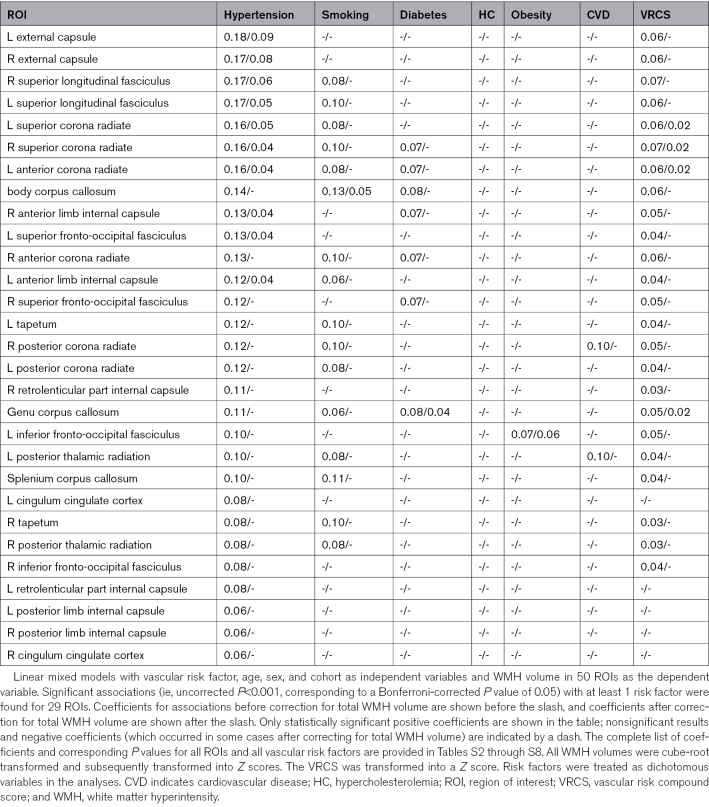
Associations Between Individual Vascular Risk Factors and Regional WMH Volumes

**Table 5.**
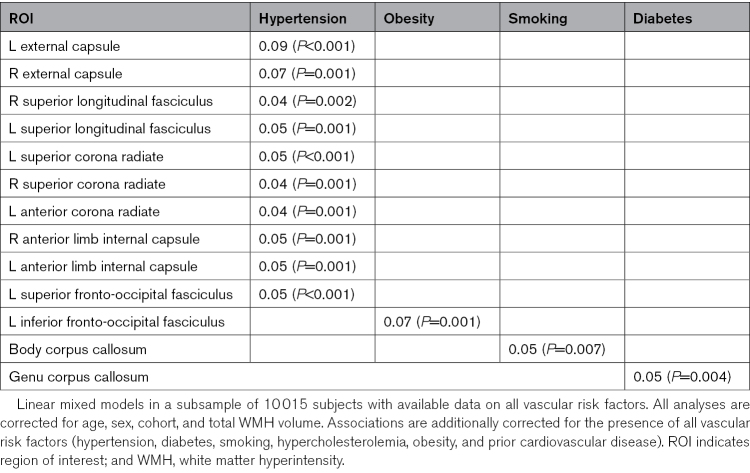
Association Between Hypertension and Obesity, and Regional WMH Volumes After Additional Correction for Presence of Other Vascular Risk Factors

**Figure.**
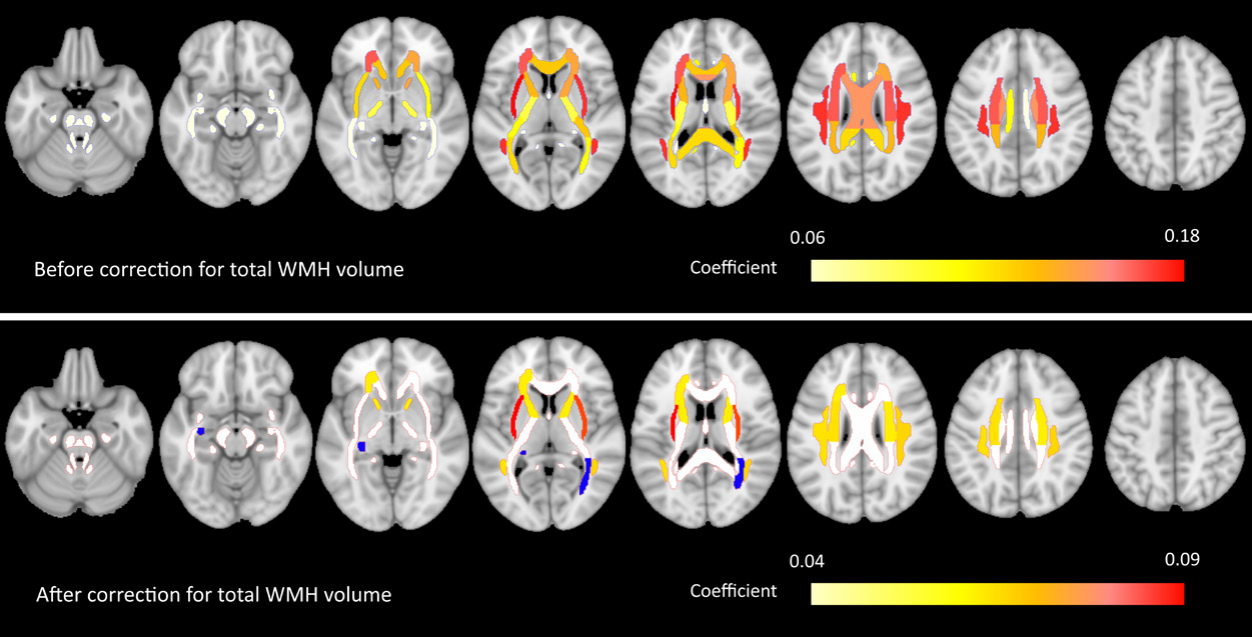
**White matter regions associated with hypertension.** Visualization of results of the linear mixed models in which hypertension was associated with white matter hyperintensity (WMH) volume in 50 regions. **Top**, The results before correction for total WMH volume. **Bottom**, The results after correction for total WMH volume. All results are corrected for age, sex, and study site. Significant regions of interest (ROIs) are color-coded based on their coefficient. ROIs with no significant associations are shown in white. Two ROIs were significantly inversely associated with hypertension after correction for total WMH volume (fornix and striae terminalis on the left side and posterior thalamic radiation on the right side) and are shown in blue. The left hemisphere is depicted on the left.

Obesity was associated with one region (the left inferior fronto-occipital fasciculus), which remained significant after additional correction for total WMH volume. Smoking was associated with WMH volume in 15 regions, one of which remained significant after correction for total WMH volume (body corpus callosum), diabetes with 7 regions, one of which remained significant after correction for total WMH volume (genu corpus callosum), and prior cardiovascular disease with 2 regions, none of which remained significant after correction for total WMH volume. For hypercholesterolemia, no significant regions were found, neither before nor after correction for total WMH volume.

The VRCS was significantly associated with WMH volume in 24 regions, 4 of which (bilateral superior corona radiata, left anterior corona radiata, and genu corpus callosum) remained significant after correction for total WMH volume. All significant regions for VRCS were also associated with hypertension, whereas several regions that were associated with hypertension were not associated with the VRCS.

#### Post Hoc Age-Stratified Analyses

Hypertension significantly interacted (*P*<0.05) with age in relation to regional WMH volume in all 10 significant models. In stratified analyses (shown in Table S9 and Figure S3), overall coefficients were higher in individuals aged 60 to 80 years (n=9796; coefficients ranging from 0.05 to 0.10) compared with individuals aged <60 years (n=4888; coefficients ranging from 0.01 to 0.06). In individuals aged >80 years (n=848), the coefficients were relatively high in relation to the left (B=0.31; *P*=0.001) and right (B=0.33; *P*<0.001) external capsule, and left (B=0.15; *P*=0.028) and right (B=0.26; *P*<0.001) superior longitudinal fascicle, whereas coefficients for the corona radiata, anterior limb of the internal capsule, and left superior fronto-occipital fasciculus were lower and not statistically significant. After restandardizing *Z* scores of WMH volumes within each age stratum, the coefficients in individuals aged <60 years became larger than the coefficients in individuals aged 60 to 80 years (Table S10; Figure S3).

Obesity significantly interacted (*P*=0.003) with age in relation to regional WMH volume in the left inferior fronto-occipital fasciculus. In stratified analyses, overall coefficients were higher in individuals aged 60 to 80 years (n=9279; B=0.08; *P*<0.001) compared with individuals aged <60 years (n=4643; B=0.03; *P*=0.05) and highest in individuals aged >80 years (n=715; B=0.12; *P*=0.293). After restandardizing *Z* scores of WMH volumes within each age stratum, coefficients were comparable in individuals aged <60 years (B=0.06) and 60 to 80 years (B=0.07) and highest in individuals aged >80 years (B=0.10). Diabetes significantly interacted with age (*P*<0.001) in relation to regional WMH volume in the genu of the corpus callosum. In stratified analyses, overall coefficients were slightly higher in individuals aged 60 to 80 years (n=9709; B=0.04; *P*=0.002) compared with individuals aged <60 years (n=4860; B=0.03; *P*=0.068) and highest in individuals aged >80 years (n=838; B=0.05; *P*=0.342). After restandardizing *Z* scores of WMH volumes within each age stratum, coefficients were comparable across age strata (<60 years: B=0.05; 60–80 years: B=0.04; and >80 years: B=0.04). Smoking did not interact with age in relation to WMH volume in the body of the corpus callosum.

In the sensitivity analyses including only individuals without a history of prior stroke, the main results were unchanged (Table S11).

## Discussion

In this large, multicenter population-based study, we identified signature WMH locations associated with vascular risk factors. We found that hypertension was most strongly related to total WMH volume, with discrete signature WMH locations, mainly involving anterior white matter regions, more specifically the bilateral external capsule, superior longitudinal fasciculus, superior corona radiata, anterior limb of the internal capsule, left anterior corona radiata, and left superior fronto-occipital fasciculus. Associations between hypertension and WMH volume in these regions remained significant after correction for total WMH volume and other vascular risk factors, indicating that these associations are region-specific and are not explained by a global increase in WMH or the presence of other vascular risk factors. Smoking (body corpus callosum), diabetes (genu corpus callosum), and obesity (left inferior fronto-occipital fasciculus) were each associated with one region after correction for total WMH volume. There was no overlap between the 13 regions associated with these 4 risk factors (ie, each region was associated with only a single risk factor after correction for total WMH volume). We found no signature WMH locations (ie, associations independent of total WMH volume) for hypercholesterolemia and history of cardiovascular disease, suggesting that these risk factors exert more global effects on WMH.

Concerning global WMH burden, our multivariate analysis of the relation between 6 vascular risk factors and total WMH volume shows that hypertension, smoking, and diabetes are each independently associated with total WMH volume, which is in line with several prior studies.^13,20,27,28^ Our findings suggest that, in addition to age, hypertension is the strongest determinant of global WMH burden. Several prior studies addressed the relation between vascular risk factors and WMH location (Table 1). A direct comparison of these studies is complicated by heterogeneity in design and methodology. Study populations varied, for example, memory clinic patients with subjective cognitive decline, mild cognitive impairment, or dementia, asymptomatic individuals recruited from the population, or individuals who had a stroke. Hypertension was most frequently studied. Two studies only used a VRCS. Most studies used ROI-based analyses, with almost no overlap between atlases or parcellations used, and several studies used a voxel-based approach. ROI- and voxel-based approaches each have distinct advantages and limitations. The main advantage of a voxel-based approach is that it provides high spatial resolution, at the level of individual voxels of for example 1×1×1 mm, with the main limitations being the need to correct for multiple comparisons in many thousands of voxels and challenges in achieving a sufficient lesion coverage (ie, each voxel needs sufficient individuals with a lesion to be included in the analyses). In contrast, the main advantage of ROI-based analyses is that grouping of voxels into regions reduces the number of statistical tests (requiring a less strict correction for multiple comparisons) and increases statistical power but comes with the disadvantage of potentially overlooking effects that are highly localized in small sections of a specific ROI, as these effects may be diluted. Some prior studies corrected for total WMH volume (which is required to rule out that regional effects are explained by an increase in total rather than regional WMH volume), whereas others did not. The overall pattern that emerges from these studies is that, across all clinical domains, hypertension was most consistently associated with anterior (ie, frontoparietal) WMH. Prior studies on other vascular risk factors provided conflicting results, with some studies reporting specific WMH locations for smoking, diabetes, or obesity, which were not reproduced in the other studies. With our study, the largest to date, we were able to include 6 vascular risk factors, and to use a detailed anatomic parcellation of the white matter including 50 ROIs, thus providing high spatial resolution compared with most prior studies (see Table 1 for a comparison). Our findings (1) confirm that hypertension is associated with anterior WMH, (2) provide a detailed map of white matter regions associated with hypertension, including effect sizes that pinpoint the regions where these associations are strongest, (3) show that other risk factors have a limited (obesity, smoking, and diabetes) or no (hypercholesterolemia and history of cardiovascular disease) region-specific effect on the occurrence of WMH; nevertheless, our finding that obesity, smoking, and diabetes were each associated with WMH in one unique region of the white matter is interesting and merits further research, (4) show that associations between the VRCS and WMH location are mainly driven by hypertension, and (5) our age-stratified analyses suggest that the region-specific effects of hypertension on WMH occurrence may be age-dependent (Tables S9 and S10), with the largest effect on WMH volumes in the external capsule and superior longitudinal fasciculus in individuals aged >80 years, also after restandardization of effect sizes within age strata.

The region-specific association between hypertension and WMH suggests that certain white matter regions, that is, the anterior white matter, are particularly vulnerable to the effects of hypertension, whereas other white matter regions are more resilient. A possible explanation is that blood pressure in cerebral arterioles is not uniform^29^ but decreases as a function of their length and the number of branches.^20,30^ Lenticulostriate arterioles, which supply anterior periventricular and peribasal ganglia white matter, are relatively short, have few branches, and are, thus, exposed to high blood pressures (almost as high as in the carotid artery).^30^ High blood pressure in these short arterioles may predispose to arteriolosclerosis and lipohyalinosis and contribute to WMH formation. In contrast, arterioles that supply the subcortical white matter are longer and have more branches, resulting in lower blood pressures.^30^ In a mathematical modeling study, systolic blood pressures in the posterior parietal arteriolar beds were estimated to be ≈30% lower compared with lenticulostriate arteriolar beds.^30^ Lower blood pressure in arterioles that are longer and have more branches may offer protection against the effects of hypertension, possibly at the cost of a higher risk of hypoperfusion in the case of hypotension, especially if hypotension is combined with pathology of large vessels, for example, arterial stiffening and corresponding wide pulse pressures.^30^ Of note, theories about differential blood pressures as a function of the length of cerebral arterioles are mainly based on mathematical models, and studies that have measured blood pressures in cerebral arterioles in humans are scarce.^30^ Furthermore, WMH on brain MRI is not a homogeneous entity but instead reflects several possible neuropathological changes with a complex and diverse pathophysiology.^3,6^ The most straightforward mechanism for the link between hypertension and WMH is arteriolosclerosis of the cerebral small vessels, as discussed above.^6,31^ However, other factors including, for example, blood-brain barrier leakage, atherosclerosis of large vessels (eg, the carotid arteries), and cardiac disease may be promoted by hypertension and may contribute to cerebral hypoperfusion, ischemia, and WMH formation.^3,31,32^ Studies with a longitudinal design are needed to further substantiate the notion that anterior white matter is more susceptible to the effects of hypertension. Furthermore, future studies may benefit from the use of detailed markers for small vessel function (eg, high-resolution imaging for small vessel function, blood-brain barrier leakage, and regional brain metabolism) and markers for systemic vascular disease mechanisms (eg, using blood biomarkers) to identify the mechanisms involved in region-specific susceptibility of the white matter to the effects of hypertension. Such studies may help elucidate the pathways for hypertension-related white matter injury and ultimately may provide treatment targets to ameliorate these effects.

It is possible that the effects of vascular risk factors on WMH may differ across the lifespan. Because the age of included individuals ranged from 18 to 97 years, we performed post hoc analyses of our main significant results by adding interaction terms (vascular risk factor*age) to the model, followed by a stratification for age in the case of a significant interaction. The reason for performing these analyses before and after restandardization of the *Z* scores was to offer a fairer comparison of effects within and between age strata. Because WMHs are strongly age-dependent, *Z* scores before restandardization are larger in the old, apparently inflating effect sizes. Restandardization provides an indication of the relative effect of the vascular risk factor within each age stratum. This, indeed, resulted in increased coefficients in the young and attenuated coefficients in the very old (see the Results section, Tables S9 and S10, and Figure S3; also note that restandardization does not affect *P* values, only effect sizes). These post hoc analyses showed that the association between hypertension and obesity, and total WMH volume was strongest (ie, coefficients were largest, also after restandardization) in older individuals (aged 60–80 years) compared with young (<60 years) and very old (>80 years) individuals. A similar pattern was observed for the association between hypertension and obesity, and regional WMH volumes, with 2 notable exceptions. First, after restandardizing the *Z* scores for WMH volumes within each age stratum, coefficients for hypertension in relation to regional WMH volumes were higher in individuals aged <60 years than in individuals aged 60 to 80 years (whereas the reverse pattern was observed for total WMH volume). Second, the association between hypertension and WMH volume in the external capsule and superior longitudinal fasciculus was strongest in the very old, with coefficients up to 2× larger than at younger ages. This finding may suggest that these white matter regions may be particularly vulnerable to hypertension in the very old. However, given the post hoc nature of these stratified analyses, corroboration in an independent cohort is warranted before any strong conclusions can be drawn.

Strengths of our study are the large sample size, multicenter setting with extensive geographic coverage, detailed anatomic parcellation of the white matter (using 50 regions), and rigorous correction for multiple comparisons. The large sample size, with a fair representation of different age strata, enabled the stratification for age in the case of significant interaction terms. The main limitation is the cross-sectional design, which means that the observed associations between vascular risk factors and WMH may not reflect causal relations. Furthermore, there was limited ethnic diversity in the included study participants (ie, 83.8% were white), which may limit generalizability to nonwhite populations. There was heterogeneity in image acquisition and processing. However, we have previously shown that despite this heterogeneity, the harmonized data on WMH were sufficiently homogeneous to justify the merging of cohorts.^2^ There was also heterogeneity regarding definitions for vascular risk factors across cohorts. For example, hypertension was defined based on a combination of blood pressure, use of antihypertensive medication, and self-reported history of hypertension in most studies. We corrected all analyses for the study site to minimize the impact of cohort-specific effects on the results. We used dichotomous variables for vascular risk factors, which may provide lower statistical power compared with using continuous variables. Data on duration of hypertension and other risk factors were not available and could, therefore, not be considered in the analyses. The use of antihypertensive, lipid-lowering, and antidiabetic medication may have influenced the associations between hypertension, hypercholesterolemia, or diabetes and WMH volumes. The current data set did not permit detailed analyses of such potential influences. The correction of associations between individual vascular risk factors and regional WMH volumes for total WMH volume could theoretically have introduced a collider bias, possibly resulting in underestimation of coefficients. We attempted to correct for possible collider bias by additionally correcting models for all other vascular risk factors (Table 5; results essentially unchanged), but we cannot rule out residual effects of collider bias. Furthermore, it should be noted that WMH volume in each of the 50 included regions was also included in total WMH volume, resulting in a small amount of overlap in regional and global WMH volumes. Finally, because of the strict correction for multiple comparisons (ie, the Bonferroni correction for analyzing 50 ROIs, meaning that an uncorrected *P* value of 0.001 was used as a threshold for statistical significance), more subtle associations may have been overlooked. We prioritized specificity over sensitivity and emphasized effect sizes, which are not affected by the Bonferroni correction, instead of only focusing on *P* values.

In conclusion, hypertension has a signature WMH pattern, involving anterior white matter regions. Associations between other vascular risk factors and regional WMH volumes are mainly explained by a global increase in WMH, rather than reflecting regionally specific effects. The specific vulnerability of anterior white matter regions to the effects of hypertension merits further research and may provide novel treatment targets for the prevention of hypertension-related white matter injury.

## Article Information

### Acknowledgments

The authors thank the study participants for their participation in ASPS (Austrian Stroke Prevention Study), ASPSF (Austrian Stroke Prevention Study Family), AUCD (Alzheimer's Disease UC Davis Diversity Cohort), CNS (Calgary Normative Study), CU-RISK (Chinese University of Hong Kong Risk Index for Subclinical Brain Lesions in Hong Kong Study), EDIS (Epidemiology of Dementia in Singapore Study), FHS (Framingham Heart Study), HCHS (Hamburg City Health Study), LBC1921 (Lothian Birth Cohort 1921), LBC1936 (Lothian Birth Cohort 1936), MAS (Sydney Memory and Ageing Study), OATS (Older Australian Twin Study), RS (Rotterdam Study), and SABRE (Southall and Brent Revisited Study) and also members of the scientific and data collection teams who have been involved in the data collection. Dr Biesbroek was involved in conceptualization, methodology, validation, investigation, formal analysis, visualization, writing-original draft, project administration, and funding acquisition. Dr de Kort was involved in conceptualization, methodology, validation, investigation, data curation, project administration, and writing-review and editing. Dr Biessels was involved in conceptualization, supervision, writing-review and editing, and funding acquisition. All other authors were involved in resources, data curation, and writing-review and editing.

### Sources of Funding

This work was supported by Veni Grant 9150162010055 from the Netherlands Organisation for Health Research and Development (ZonMw) to Dr Biesbroek. Harmonization of data through the Meta VCI Map Consortium was supported by Vici Grant 918.16.616 from the Netherlands Organisation for Health Research and Development (ZonMw) to Dr Biessels. This research is part of the Timely, Accurate, and Personalized Diagnosis of Dementia (TAP-Dementia) program, which receives funding from the Netherlands Organisation for Health Research and Development (ZonMw; grant 10510032120003). Dr Beiser and S. Seshadri were supported by the National Institutes of Health (NIH) grants R01 AG 054076, R01 AG 033040, R01 AG 016495, and U19 NS 120384. Drs Cheng, Thomalla, Petersen, and Mayer were supported by Deutsche Forschungsgemeinschaft (grant SFB 936), project number 178316478, Project C2. Dr DeCarli was supported by NIH grants P30 AG072972, U19 NS 120384, UF1NS100608, and R01 AG 031563. Drs McCreary and Frayne were supported by the Canadian Institutes of Health Research Foundation Award. Dr Fletcher was supported by NIH grant R01 AG 031563. Dr Maillard was supported by NIH grant UF1NS100608. Dr Wardlaw was supported by the UK Dementia Research Institute Ltd (Edin002, DRIEdi17/18, Medical Research Council [MRC] MC_PC_17113), which receives its funding from DRI Ltd, funded by the UK Medical Research Council, Alzheimer’s Society, and Alzheimer’s Research UK; The Row Fogo Center for Research into Ageing; the Brain (AD.ROW4.35.BRO-D.FID3668413); Age UK; and MRC grants G0701120 and G1001245. Dr Cox is supported by a Sir Henry Dale Fellowship jointly funded by the Wellcome Trust and the Royal Society (grant 221890/Z/20/Z), Age UK (The Disconnected Mind project), the UK Medical Research Council (grant MR/R024065/1), the Milton Damerel Trust, and joint funding from the UK Biotechnology and Biological Sciences Research Council and the Economic and Social Research Council (grant BB/W008793/1). Dr Vinke was supported by the Dekker Postdoc Grant from the Dutch Heart Foundation (grant 03-006-2023-0077). Dr Vernooij is a recipient of ABOARD, which is a public-private partnership receiving funding from the Netherlands Organisation for Health Research and Development (ZonMw; grant 73305095007) and Health~Holland, Topsector Life Sciences & Health (PPP-allowance; grant LSHM20106). Dr Vernooij further received funding from the Netherlands Organisation for Health Research and Development (ZonMw) Memorabel Grant (733050817) and TAP-Dementia, the Netherlands Organisation for Health Research and Development (ZonMw)–funded project (grant 10510032120003) in the context of the Dutch National Dementia Strategy. Dr Kuijf was supported by the Dutch Heart Foundation project 03-004-2021-T043. Dr Chaturvedi is supported by the MRC grant MC_UU-00019/1. H. Brodaty received funding from the National Health and Medical Research Council. Sources of funding for individual cohorts are provided in the Supplemental Material.

### Disclosures

Dr Sachdev has served on the Advisory Committees of Biogen Australia and Roche Australia from 2020 to 2022 and Eli Lilly in 2025. S. Seshadri received consulting fees and speaker honoraria from Eisai and Lilly. In addition, S. Seshadri serves on a scientific advisory board for Lilly. Dr Chaturvedi serves on Data and Safety Monitoring Boards for AstraZeneca. Dr Sudre is a scientific advisor to BrainKey. H. Brodaty is or has been an advisory board member or a consultant to Biogen, Eisai, Eli Lilly, Medicines Australia, Roche, Skin2Neuron, and Cranbrook Care. Dr de Groot reports stock ownership and previous employment with GlaxoSmithKline; GlaxoSmithKline had no role in this study. Dr DeCarli reports compensation from Novo Nordisk for consultant services. Dr Muñoz Maniega reports grants from the Biotechnology and Biological Sciences Research Council. The other authors report no conflicts.

### Supplemental Material

Supplemental Methods

Tables S1–S11

Figures S1–S3

Sources of Funding for Individual Cohorts

References 33–49

## Supplementary Material


