# Supplementary material for: Signature White Matter Hyperintensity Locations Associated With Vascular Risk Factors Derived From 15 653 Individuals
Source: Stroke. 2025 Aug 15;56(10):3047–59. doi: 10.1161/STROKEAHA.125.051159 (PMC12447828; doi:10.1161/STROKEAHA.125.051159)
Supplement: Supplementary file 1 [file str-56-3047-s001.pdf]

SUPPLEMENTAL MATERIAL

Signature white matter hyperintensity locations associated with specific cardiovascular risk factors derived from 16 population-based cohorts comprising 15,653 individuals

Supplemental methods

- Table S1. Cohort-specific details
- Harmonization methods
- Image processing
- Figure S1. Flowchart of participant selection

Please note that the supplemental methods were almost entirely reproduced from the supplements of the following prior publication by de Kort et al.[2] [de Kort et al, Cerebral white matter hyperintensity volumes: Normative age- and sex-specific values from 15 population-based cohorts comprising 14,876 individuals, is licensed under CC BY 4.0].

Supplemental results

- Figure S2. WMH prevalence map
- Table S2. Linear mixed models for hypertension
- Table S3. Linear mixed models for smoking
- Table S4. Linear mixed models for diabetes mellitus
- Table S5. Linear mixed models for hypercholesterolemia
- Table S6. Linear mixed models for obesity
- Table S7. Linear mixed models for history of cardiovascular disease
- Table S8. Linear mixed models for the vascular risk compound score
- Table S9. Association between hypertension and regional WMH volumes, stratified for age
- Table S10. Association between hypertension and regional WMH volumes, stratified for age, after restandardizing WMH volumes.
- Table S11. Sensitivity analyses: associations between vascular risk factors and regional WMH volumes after excluding patients with a history of prior stroke.
- Figure S3.

Sources of funding for individual cohorts

Table S1: Cohort-specific details

Cohort	Study design	Population	Recruitment strategy	Recruitment period	Age criterium	Other main inclusion criteria	Main exclusion criteria	Response rate study	Sample size at baseline	Participants invited for brain MRI; response rate MRI	Population/ datapoints used in current study
Austrian Stroke Prevention Study - Original (ASPS) [34]	prospective single-center population based	population of Graz, Austria	- a random population sample, via the official community register, received a written invitation to participate<br>- balanced distribution based on sex and 5-year age groups	1991-1994	50-75 years	none	- history of neuropsychiatric disease, including stroke and dementia<br>- abnormal neurologic examination at structural clinical examination	34% interested and eligible	1998 individuals (enrollment stopped after inclusion of 1998 individuals)	every 4th participant; 92%	baseline MRI and corresponding clinical data. Follow-up MRI and data were used in case of missing or bad quality FLAIR, and no overlap with ASPSF
Epidemiology of Dementia in Singapore Study (EDIS) [35]	Prospective single center population-based	Population of Singapore	- First phase: participants from the Singapore Epidemiology of Eye Disease Study (a population-based study), aged ≥60 years, were screened for possible cognitive decline based on either an abnormal Abbreviated Mental Test or reported cognitive decline by caretaker<br>- Screen-positive individuals (n=1598) were invited for the second phase which included neuropsychological testing and brain MRI	2010-2015	≥60 years	none	none	N/A	957 individuals participated in the second phase	All enrolled; 100%	Baselin MRI and corresponding clinical data
Austrian Stroke Prevention Family Study (ASPSF) [36]	prospective single-center offspring study	population of Graz, Austria	- study participants of ASPS and their first-grade relatives were asked to participate	2006-2013	50-75 years	none	- history of neuropsychiatric disease, including stroke and dementia<br>- abnormal neurologic examination at	N/A	381 individuals	all enrolled were invited; 93%	baseline MRI and corresponding clinical data

							structural clinical examination				
Alzheimer's Disease UC Davis Diversity Cohort (AUCD) [37]	prospective two-center population based	population of California, served by the University of California, Davis Alzheimer's Disease Center	-community outreach program<br>- Recruitment goals were focused on 1) a cognitively heterogeneous sample 2) of 1/3 Hispanic, 1/3 African American and 1/3 non-Hispanic Caucasian decent	2002–2007	≥ 60 years	fluent in English or Spanish	- unstable major medical illness<br>- major primary psychiatric disorder<br>- substance abuse or dependence in the last 5 years	N/A	1357 individuals completed cognitive screening with SENAS at baseline	all enrolled in the longitudinal cohort were invited; 86%	a random sample of screened individuals with no significant impairment on one of the SENAS scales (measuring episodic memory, language/ semantic memory, spatial ability, abstract reasoning, and attention span)
Calgary Normative Study (CNS) [38]	prospective single-center population based	population of Calgary, Canada	- via posters and word-of-mouth<br>-balanced distribution based on sex and 10-year age groups	inclusion started 2013, ongoing	≥ 18 years	none	- history of neuropsychiatric disease, including stroke and dementia<br>- contra-indications for MRI	N/A	ongoing	all enrolled; 100%	baseline MRI and corresponding clinical data
The Chinese University of Hong Kong - Risk Index for Subclinical brain lesions in Hong Kong Study (CU-RISK) [39]	prospective single-center population based	population of Hong Kong SAR, China	- advertisements in local community-centers and word-of-mouth	2011-2015	≥ 65 years	- functional independence as defined by a score of 20 on the 20-point Barthel Index and < 2 on the Lawton's Instrumental of Daily Living Scale (IADL)<br>- Cantonese-speaking<br>- sufficient sensorimotor and language competency for cognitive testing	- history of neuropsychiatric disease, including stroke, TIA and dementia<br>- evidence of brain tumors, large cerebral infarcts (i.e., infarcts ≥20 mm in diameter), cortical infarcts, or hydrocephalus on MRI	N/A	830 individuals	all enrolled; 100%	baseline MRI and corresponding clinical data

Framingham Heart Study-Offspring cohort (FHS_ Gen2) [40]	prospective single-center offspring study	population of Framingham, USA	- biological children and their spouses of the Original FHS-cohort members were asked to participate	inclusion started 1971; health examinations ± every 4 years	12-60 years, at baseline	none	- history of neuropsychiatric disease, including stroke and dementia	Ca. 80%	5124 individuals attended first round of gen II	all attendees of exam 8 (n=3021) were invited; 51%	exam 8 and corresponding MRI (2005-2008)
Framingham Heart Study- third generation cohort (FHS_ Gen3) [41]	prospective single-center offspring study	population of Framingham, USA	-adults with at least one parent in the Offspring Cohort received invitation letters<br>-priority given to extended families	first round 2002-2005	19-72 years, at baseline	none	- history of neuropsychiatric disease, including stroke and dementia	62,5% interested and eligible	4095 individuals attended first round of gen III	all attendees of exam 2 (n=3411) were invited; 59%	exam 2 and corresponding MRI (2009-2011)
Framingham Heart Study-OMNI-I cohort (FHS_ OMNI1) [42]	prospective single-center population based	population of Framingham, USA	Framingham residents who self-identified as members of a minority group (African American, Hispanic, Asian, Indian, Pacific Islander and Native American descent)	inclusion started 1994	40-74	none	- history of neuropsychiatric disease, including stroke and dementia	N/A	506 participants attended first round of OMNI-I	all attendees of exam 3 (n=298) were invited; 48%	exam 3 and corresponding MRI (2005-2008)
Hamburg City Health Study (HCHS) [43]	prospective single-center population based	population of Hamburg, Germany	- a random sample from the official inhabitant data file received written invitation letters<br>-divided into six age and gender strata	2016-ongoing.	45–74 years	none	none	N/A	enrollment ongoing at this time. First round (2016-2018) included 10.000 individuals	subgroup of 2,657 from 10.000 participants selected for brain MRI	baseline MRI and corresponding clinical data
Lothian Birth Cohort 1921 (LBC1921) [44], [45]	observational longitudinal, population based	population of the Lothian area, Scotland	- individuals born in 1921 who lived in the Lothian area of Scotland at recruitment, identified using the Community Health Index, received written invitation letters (including one reminder), followed by media advertisement	1999-2001	birth-year 1921	- availability of test results of the Scottish Mental Survey of 1932	none	49,1% of all invited individuals were included at wave 1	550 individuals were included at wave 1	brain imaging was included in wave 5; all enrolled invited; 90%	wave 5 (2013)

Lothian Birth Cohort 1936 (LBC1936) [46]	observational longitudinal, population based	population of the Lothian area, Scotland	- individuals born in 1936 who lived in the Lothian area of Scotland at recruitment, identified using the Community Health Index, received written invitation letters (including one reminder), followed by media advertisement	2004-2007	birth-year 1936	- availability of test results of the Scottish Mental Survey of 1947	none	29,6% of all invited individuals were included at wave 1	1091 individuals were included at wave 1	brain imaging was included in wave 2; all enrolled invited; 84%	wave 2 (2007-2010)
Sydney Memory and Ageing Study (MAS)[47]	prospective single-center population based	population of Sydney, Australia	- a random population sample through the electoral roll from two federal government areas, participants received invitation letters	2005-2007	70-90	- availability of an informant who had to have at least weekly contact of not less than one hour with the participant	- history of neuropsychiatric disease, including dementia, progressive malignancy or psychotic symptoms<br>- medical or psychological conditions that may have prevented them from completing assessments<br>- MMSE score of < 24	19,9% interested	1037 individuals attended baseline assessment	all enrolled were invited; 53%	wave 1 (baseline)

Older Australian Twins Study (OATS)[48]	prospective single-center	twins and their siblings, New South Wales, Victoria and Queensland, Australia	- twins were contacted through the Australian Twin registry, media release and newspaper advertisement.	2006-2012	>65 years	- having a consenting co-twin<br>- having completed some education in English<br>- at least low average intelligence (IQ ≥ 80).	- diagnosis of malignancy or other life-threatening illness or acute psychosis-inadequate English to participate in assessments	N/A	623 individuals (600 twins, 23 siblings) attended baseline assessment	all enrolled were invited; 66%	wave 1 (baseline)
Rotterdam Study (RS)[49]	prospective single-center population based	population of Ommoord, Rotterdam, The Netherlands	- a random population sample, participants received invitation letters	RS-I: 1990-1993<br>RS-II: 2000<br>RS-III: 2006	RS-I and RS-II: ≥55 years<br>RS III: ≥ 45 years	none	none	combination of RS-I, RS-II and RS-III at baseline: 72%	combination of RS-I, RS-II and RS-III at baseline: 14,926 individuals	all enrolled were invited;<br>RS-I-5: 81%<br>RS-II-3: 80%<br>RS-III-2: 75%	combination of waves RS-I-5, RS-II-3, RS-III-2; all with availability of MRI (protocol > 2005) and corresponding clinical data
Southall and Brent Revisited Study (SABRE) [50]	follow-up population based cohort study	population of West London, UK	- for the original Southall and Brent cohort studies, participants were recruited from either their workplaces (20%) or randomly selected from general practice registers (80%).<br>- at wave 3, partners of each index participant were invited and new recruitment of African Caribbean participants was carried out by clinic staff who went into local communities.	original cohort studies: 1988-1991<br>SABRE follow-up study: 2008-2012<br>Wave 3: 2014-2018	original cohort studies: 40-69 years<br>SABRE follow-up study:<br><br>Wave 3: Index: 65-90 years, new participants: 37-90 years	None	None	original cohort studies at baseline (combined): 63%<br>Wave 3: N/A	original cohort studies at baseline (combined): 4972 individuals<br>SABRE: 1438 individuals<br>Wave 3: 1000 individuals (Index: 654, new partners: 249 and new African Caribbean: 97)	all enrolled were invited; 78%	wave 3 (2014-2018)

Harmonization methods
Given the heterogeneity in data collection methods employed by the different cohorts, the data required harmonization steps. Below, a more detailed description and justification of the variable recoding is given. This harmonization procedure, with the exception of the harmonization of the EDIS cohort, has been previously described elsewhere.[2]
Variables for ***Sex and Body Mass Index*** could be unambiguously recoded to confirm the definitions specified below.

Schematic representation of variables unambiguously recoded

Variable	Definition	Categories/ units
Sex	Sex assigned at birth	Male / female
BMI	Body Mass Index	kg/m^2

Age
Age was defined as the chronological age in years at time of imaging. For participants of the SABRE cohort, only the birth year was provided. As all participants were evaluated between 2014 and 2016, the age was approximated by the difference between birth year and 2015.

Race and ethnicity
Based on availability of data, race and ethnicity was recoded into four categories: White, Black, Asian and Other. The categories included in the "other" group were too small to make separate categories, however, to be as specific as possible, details on the races and ethnicities included in this group are provided below. For the LBC1936, LBC1921, ASPS, ASPSF and HCHS all individuals were Caucasian White and were labeled as White. For the RS, 97% of individuals were assumed to be White.

Schematic representation of the recoding of race

Cohort	Asian	Black	White	Other
CNS	Asian	Black	White	Hispanic, First Nations
MAS/ OATS	Asian	African	Caucasian	Indigenous Australian, Torres Strait Islander, Pacific Islander, Mixed
SABRE	South-East Asian	African Caribbean	European	Other
AUCD	Asian	African American	White	Pacific Islander, Filipino, Hispanic, Other
FHS	Asian	Black	White	Hispanic or Latino, Native Hawaiian/Pacific Islander, American Indian/Alaskan Native, Asian Indian/Pacific Islander, Multiracial
EDIS	Indian, Malay, Chinese	N/A	N/A	N/A

Definitions for vascular risk factors

Smoking status
Smoking status was harmonized into 3 categories: current, past and never. HCHS defined only two categories of smoking habits, i.e. current and past smoking. EDIS defined two

categories of smoking, i.e. never versus ever, with the latter including both current and past smokers. For the current project, smoking was recoded to current smoker yes/no. Consequently, the categories past and never were merged and recoded to ‘not a current smoker’. For EDIS, participant who never smoked were recoded to ‘not a current smoker’, and participants who ever smoked were excluded because it was not clear if they were current of past smokers.

Hypertension
No universal definition could be used. Instead, the following local definitions were used to create a category with binary outcome (yes/no).

ASPS/ ASPSF
Three blood pressure measurements >160/95 mmHg or
the use of antihypertensive medication.

AUCD
Self-reported history of hypertension.

CNS
A self-reported history of hypertension or
the self-reported use of antihypertensive medication.

CU-RISK
Blood pressure measurements >140/90 mmHg or
the use of antihypertensive medication.

FHS
Systolic blood pressure ≥140mmHg or diastolic blood pressure ≥90 mmHg or
the use of antihypertensive medication

HCHS
A blood pressure measurement >140/90 mmHg,
the use of antihypertensive medication or
a self-reported history of hypertension.

LBC1921/ LBC1936
Self-reported history of ever being treated for high blood pressure

MAS/OATS
Repeated blood pressure measurements with a mean >140/90 mmHg or
the use of antihypertensive medication.

RS
Two blood pressure measurements with a mean systolic blood pressure of >140 mmHg or a mean diastolic blood pressure of >90 mmHg or
the use of antihypertensive medication.

SABRE
Physician diagnosed hypertension or
the self-reported use of antihypertensive medication.

EDIS
Systolic blood pressure ≥140 mm Hg and/or diastolic blood pressure ≥90 mm Hg, or use of antihypertensive medication

Diabetes
No universal definition could be used for diabetes. Instead, the following local definitions were used to create a category with binary outcome (yes/no).

ASPS/ ASPSF
A fasting glucose >140 mg/dL or
the use of antidiabetic medication.

AUCD
A self-reported history of diabetes.

CNS
A self-reported history of diabetes or
the use of antidiabetic medication.

CU-RISK
A fasting glucose >6,0 mmol/L,
HbA1c >5,7% or
the use of antidiabetic medication.

FHS
fasting plasma glucose of ≥126 mg/dL, random plasma glucose ≥140, or
the use of antidiabetic medication.

HCHS
A self-reported history of diabetes or
the use of antidiabetic medication.

LBC1921/LBC1936
A self-reported history of ever being diagnosed with diabetes
MAS/OATS
Physician diagnosed diabetes (not further specified).

RS
A fasting glucose of >7,00 mmol/L or
the use of antidiabetic medication.

SABRE
A fasting glucose >7,00 mmol/L or
physician diagnosed diabetes (not further specified).

EDIS
Glycated hemoglobin ≥6.5%, or use of antidiabetic drugs

Hypercholesterolemia
None of the cohorts provided blood cholesterol measurements allowing a uniform definition of hypercholesterolemia. Instead, the following local definitions were used to create a category with binary outcome (yes/no).

ASPS/ ASPSF
HDL cholesterol >40 mg/dL in men and >50 mg/dL in women or
the use of lipid lowering medication.

AUCD
A self-reported history of hypercholesterolemia

CNS
A self-reported history of hypercholesterolemia or
the use of lipid lowering medication.

CU-RISK
Elevated serum lipids (not further specified) or
the use of lipid lowering medication.

FHS
Total cholesterol and HDL levels were provided in mg/dl. The cut-off value of >240 mg/dl for total cholesterol was used to define individuals with hypercholesterolemia.

HCHS
LDL/HDL ratio >3,5 or
the use of lipid lowering medication.

LBC1921/LBC1936
A self-reported history of ever being diagnosed with high cholesterol

MAS/OATS
Physician diagnosed hypercholesterolemia (not further specified).

RS
Total cholesterol >4,14 mmol/l or
the use of lipid lowering medication.

SABRE
Use of lipid lowering medication

EDIS
Total cholesterol levels ≥4.14 mmol/L, or use of lipid-lowering medication

Cardiovascular disease
The cohorts provided details on the following forms of cardiovascular disease.

ASPS/ ASPSF
A history of symptoms of acute coronary syndrome or

signs of coronary artery disease and left ventricular hypertrophy on electrocardiogram or echocardiogram.

AUCD
A history of acute coronary artery syndrome or
previous angioplasty or coronary artery bypass graft.

CU-RISK
A history of acute coronary artery syndrome.

FHS
A history of myocardial infarction or
coronary insufficiency

HCHS
A history of myocardial infarction.

LBC1921/LBC1936
A self-reported history of ever being diagnosed with cardiovascular disease, including heart attack, angina, heart valve problems, abnormal heart rhythm or any other heart problem.

MAS/OATS
A history of acute coronary artery syndrome or peripheral arterial occlusive disease.

SABRE
A history of acute coronary artery syndrome or
Previous angioplasty or coronary artery bypass graft.

EDIS
Presence of ischemic heart disease, congestive heart failure, or cardiac bypass

Obesity
Continuous BMI values were recoded to a dichotomous variable for obesity using a cut-value of BMI≥30

Figure S1. Flowchart of participant selection

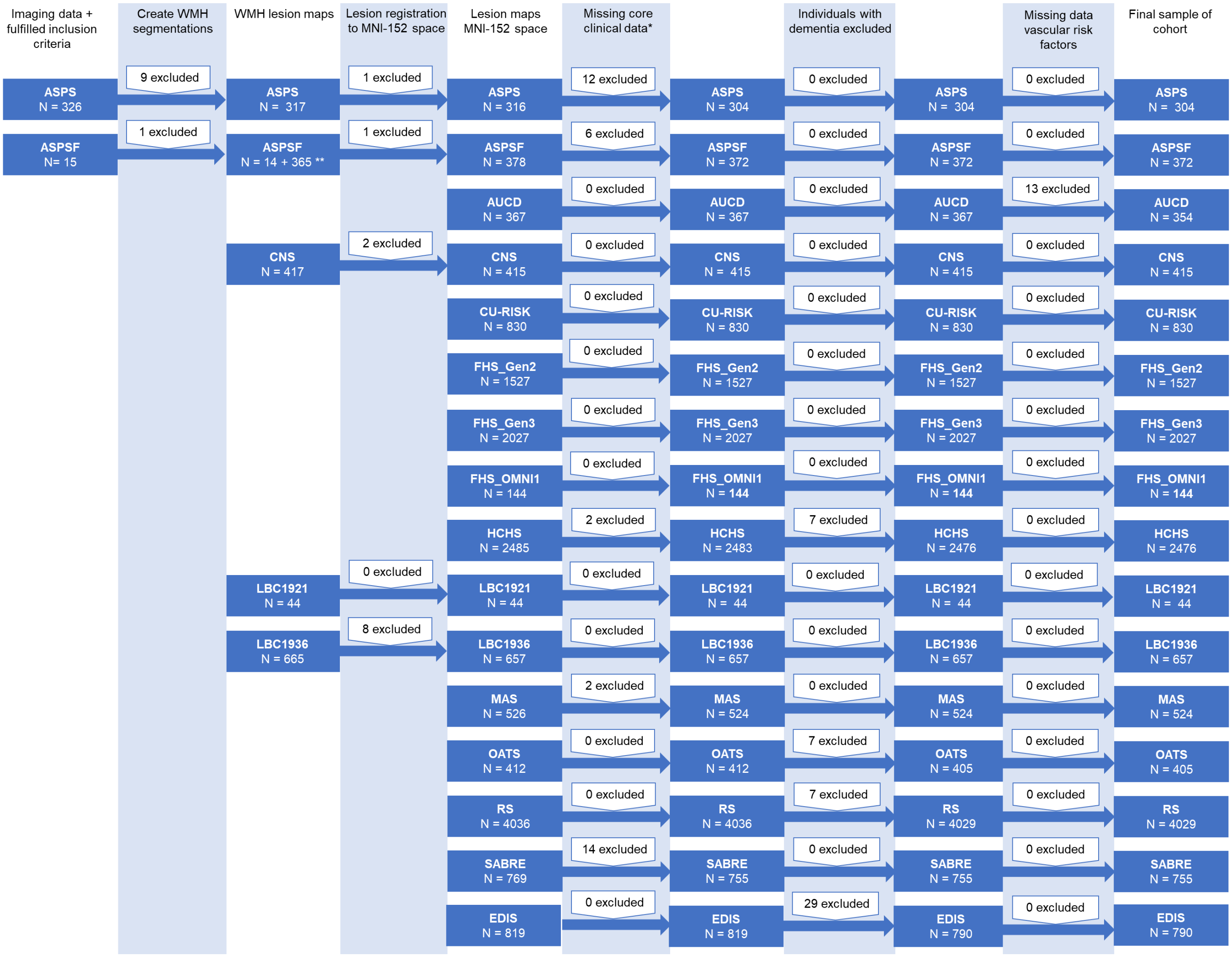

*Flowchart of participant selection stratified by cohort. Please note that the exclusion criterion 'missing data vascular risk factors' only applies when participants had missing data on all six vascular risk factors (see also section 'participant selection' in the methods in the main text). Thus, the availability of data on at least one vascular risk factor was sufficient for inclusion in the study. *Availability of clinical data on age, sex and diagnosis of dementia. **For ASPSF, 15 WMH segmentations were performed centrally and the other 365 segmentations were performed by the cohort. Abbreviations: ASPS, Austrian Stroke Prevention Study; ASPSF, Austrian Stroke Prevention Family Study; AUCD, UC Davis Alzheimer's Disease Center Diversity Cohort; CNS, Calgary Normative Study; CU-RISK, Chinese University of Hong Kong- Risk Index for Subclinical brain lesions in Hong Kong; FHS, Framingham Heart Study; Gen2, Offspring cohort; Gen3, Third generation cohort, OMNI, minorities cohort, HCHS, Hamburg City Health Study; LBC1921, Lothian Birth Cohort 1921; LBC1936, Lothian Birth Cohort 1936; MAS, Sydney Memory and Ageing Study; OATS, Older Australian Twins Study; RS, Rotterdam Study; SABRE, Southall And Brent Revisited; EDIS, Epidemiology of Dementia in Singapore Study. Figure was adapted from de Kort et al. 2024.[2] [de Kort et al, Cerebral white matter hyperintensity volumes: Normative age- and sex-specific values from 15 population-based cohorts comprising 14,876 individuals, is licensed under CC BY 4.0].*

Figure S2. WMH prevalence map

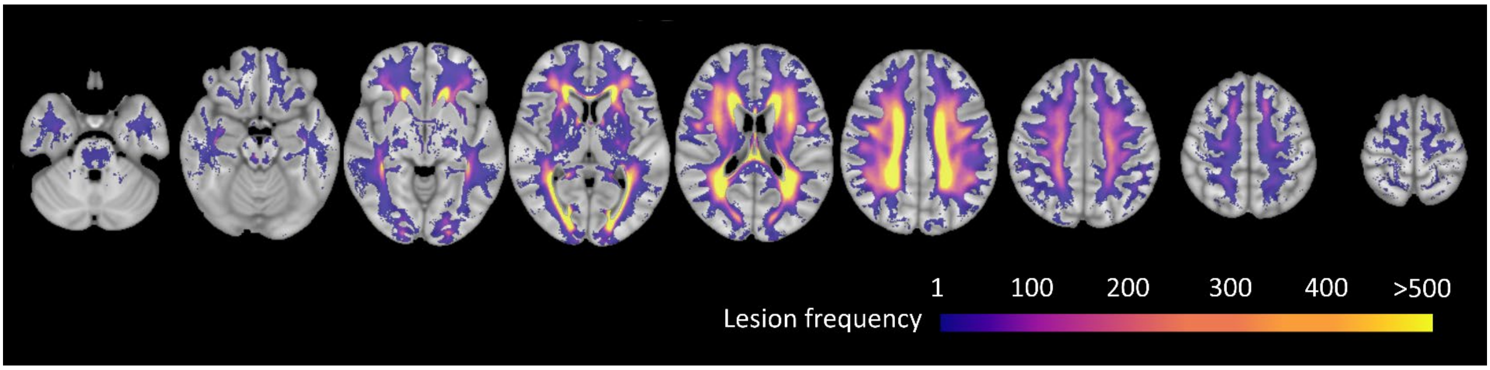

Prevalence map of white matter hyperintensities (WMH) for the total dataset of 15,653 individuals. This figure shows the number of individuals with WMH for each voxel in the brain. Displayed by radiological convention. The plasma color scale from the plasma color palette in R (version 4.1.2) was used for visualization. Abbreviations: L, Left; R, Right.

Abbreviations for regions-of-interest

ACR: anterior corona radiata
ALIC: anterior limb internal capsule
BCC: body corpus callosum
CGC: cingulum cingulate cortex
CGH: cingulum in the hippocampus
CP: cerebral peduncle
CST: corticospinal tract
EC: external capsule
FX: fornix body and column
FXST: fornix and striae terminalis
GCC: genu corpus callosum
ICP: inferior cerebellar peduncle
IFO: inferior fronto-occipital fasciculus
MCP: middle cerebellar peduncle
ML: medial lemniscus
PCR: posterior corona radiata
PCT: pontine crossing tract
PLIC: posterior limb internal capsule
PTR: posterior thalamic radiation
RLIC: retrolenticular part internal capsule
SCC: splenium corpus callosum
SCP: superior cerebellar peduncle
SCR: superior corona radiata
SFO: superior fronto-occipital fasciculus
SLF: superior longitudinal fasciculus
SS: sagittal stratum
TAP: tapetum
UNC: uncinate fasciculus

Table S2. Linear mixed models for hypertension

**Hypertension**	Before correction for total WMH volume			After correction for total WMH volume		
	Coefficient	SE	p-value	Coefficient	SE	p-value
ACR_L	0.15536213	0.01421053	**1.01458E-27**	0.04303907	0.0092004	**2.9262E-06**
ACR_R	0.13376044	0.01406192	**2.13581E-21**	0.02407487	0.00925589	0.00928804
ALIC_L	0.1242141	0.01311125	**3.08E-21**	0.04150295	0.01043347	**7.0061E-05**
ALIC_R	0.12850525	0.01357501	**3.31531E-21**	0.04292531	0.01080879	**7.2133E-05**
BCC	0.14264353	0.01451565	**1.0206E-22**	0.02400762	0.00892174	0.00716611
CGC_L	0.08214161	0.01771122	**3.58373E-06**	0.04324579	0.01731181	0.01232202
CGC_R	0.06299226	0.01777494	**0.00039788**	0.02658397	0.0174359	0.12667403
CGH_L	-0.00187197	0.01726265	0.912027934	-0.01613488	0.0172616	0.34912469
CGH_R	0.02401703	0.01718642	0.162795543	0.00923441	0.01718179	0.59158715
CP_L	-0.0015021	0.01627834	0.926398403	-0.00991782	0.01631161	0.54327099
CP_R	0.00061014	0.01665887	0.971167887	-0.00853186	0.01668959	0.60905045
CST_L	0.02456376	0.01791469	0.170967321	0.01052931	0.01792627	0.55901102
CST_R	-0.00368529	0.01796138	0.835957879	-0.0200279	0.01795413	0.26342906
EC_L	0.18026739	0.01636603	**4.26564E-28**	0.08508177	0.01359221	**4.0673E-10**
EC_R	0.17354546	0.01626514	**1.79705E-26**	0.08180701	0.01369184	**2.425E-09**
FX	0.01349343	0.01591898	0.398038012	-0.00702741	0.01584922	0.6557122
FXST_L	-0.03925351	0.01720932	0.022469206	-0.06842925	0.01702507	**5.8489E-05**
FXST_R	0.02058623	0.01747039	0.238948551	-0.0093385	0.01727432	0.58979752
GCC	0.10743804	0.0126662	**2.41241E-17**	0.0130805	0.00882677	0.13872377
ICP_L	-0.01758247	0.01816657	0.324917186	-0.02089202	0.01816449	0.24157454
ICP_R	-0.00496565	0.01773668	0.777237656	-0.00922821	0.01779149	0.60164454
IFO_L	0.09987207	0.01544071	**1.02541E-10**	0.0307607	0.01397463	0.02782784
IFO_R	0.08116631	0.01580726	**2.8743E-07**	0.00950855	0.01426742	0.50655393
MCP	-0.00063085	0.01752549	0.971664827	-0.01455568	0.01753231	0.40678791
ML_L	-0.00011192	0.01726087	0.992866153	-0.00800704	0.01730396	0.6412593
ML_R	0.00960753	0.01660147	0.564028871	0.00185309	0.01664236	0.91327043
PCR_L	0.11722601	0.01501282	**6.21247E-15**	-0.00663955	0.00909031	0.46398885
PCR_R	0.12257951	0.0150106	**3.46422E-16**	-0.00201532	0.00891136	0.82280461
PCT	-0.00481449	0.01766384	0.783885324	-0.02288422	0.01763977	0.19366697
PLIC_L	0.06320551	0.01444621	**1.22844E-05**	0.01865595	0.01384258	0.17853626
PLIC_R	0.05929145	0.0156018	**0.000146121**	0.00841601	0.01486645	0.57357691
PTR_L	0.10275176	0.01451396	**1.52233E-12**	-0.01309384	0.00924121	0.15635863
PTR_R	0.08115032	0.0143908	**1.74993E-08**	-0.03263305	0.00922962	**0.00041346**
RLIC_L	0.07780091	0.01523265	**3.33107E-07**	-0.00024386	0.01329289	0.98284738
RLIC_R	0.10780376	0.01574004	**7.80977E-12**	0.02176317	0.01340273	0.10474338
SCC	0.10154659	0.01391225	**3.08589E-13**	-0.01533008	0.008089	0.05812295
SCP_L	-0.01133509	0.01728861	0.510920774	-0.01612076	0.01734148	0.35154363
SCP_R	-0.0064835	0.01620575	0.68817769	-0.0096931	0.01625877	0.55007067
SCR_L	0.16133177	0.01438642	**4.5102E-29**	0.04602995	0.00913888	**4.821E-07**
SCR_R	0.16343939	0.01471636	**1.5103E-28**	0.04363437	0.00911057	**1.6914E-06**
SFO_L	0.12619435	0.0137343	**4.49615E-20**	0.0411001	0.01104351	**0.00019933**
SFO_R	0.11870246	0.01385032	**1.12832E-17**	0.0316975	0.01104558	0.00412054
SLF_L	0.16784395	0.01606806	**1.8965E-25**	0.05065691	0.01132087	**7.5736E-06**
SLF_R	0.17515085	0.01607632	**1.559E-27**	0.056173	0.01114452	**4.5965E-07**
SS_L	0.05068771	0.01621742	0.001797692	-0.03578164	0.01392742	0.0101396
SS_R	0.05007255	0.01560673	0.001349384	-0.0349619	0.013273	0.00844543
TAP_L	0.12280569	0.01568393	**5.28024E-15**	0.01340167	0.01155773	0.24541499
TAP_R	0.08128469	0.01439968	**1.68502E-08**	-0.0205009	0.01051673	0.05146073
UNC_L	0.03466164	0.01796098	0.054448039	0.01460966	0.017911	0.41930243
UNC_R	0.02976884	0.01783176	0.096821394	0.01560688	0.01783165	0.38730307

Linear mixed models with hypertension, age, and cohort as independent variable and WMH volume in 50 ROIs as dependent variable, before and after additional correction for total WMH volume. Uncorrected p-values are provided. To correct for multiple comparisons, a Bonferroni correction for including 50 ROIs was performed. As such, an uncorrected p-value of 0.001 was considered statistically significant. Significant p-values are shown in bold.

Table S3. Linear mixed models for smoking

**Smoking**	Before correction for total WMH volume			After correction for total WMH volume		
	Coefficient	SE	p-value	Coefficient	SE	p-value
ACR_L	0.07835661	0.02001213	**9.01925E-05**	0.00254937	0.01301844	0.84460613
ACR_R	0.09998944	0.01969606	**3.8571E-07**	0.02625824	0.01301505	0.04357042
ALIC_L	0.06084652	0.018385	**0.00093369**	0.0056641	0.01468636	0.69961543
ALIC_R	0.06222699	0.01906762	0.001099193	0.00477293	0.0152017	0.75341177
BCC	0.12908853	0.02051314	**3.16628E-10**	0.04851196	0.01266023	**0.00012734**
CGC_L	0.01782005	0.02518701	0.476812168	-0.01357109	0.02448563	0.57970957
CGC_R	0.00202112	0.0252584	0.933426004	-0.02548485	0.02469385	0.30279408
CGH_L	0.04059587	0.02454293	0.097668923	0.03022515	0.02448064	0.2163529
CGH_R	-0.00924848	0.0244572	0.706347024	-0.02031577	0.02438216	0.40509062
CP_L	-0.01630701	0.02315896	0.482976748	-0.02237311	0.02314883	0.3348881
CP_R	-0.0169418	0.02368897	0.476260758	-0.0234324	0.02367584	0.323466
CST_L	0.01808671	0.02543241	0.476166518	0.0093045	0.02539485	0.71337352
CST_R	0.04110616	0.02550485	0.106672255	0.03038075	0.02543823	0.23187569
EC_L	0.06498071	0.02324212	0.005154769	-0.00116328	0.01922357	0.94940985
EC_R	0.07083079	0.0230831	0.002141786	0.00673815	0.01932185	0.72957418
FX	0.07322961	0.02256001	0.001170969	0.05943715	0.02241096	0.0080026
FXST_L	0.04098627	0.02446956	0.093507085	0.02033603	0.02414827	0.39898629
FXST_R	0.00987219	0.02485189	0.689487434	-0.01210322	0.02449709	0.62181082
GCC	0.06112685	0.01765411	**0.00053418**	-0.00202413	0.01228982	0.86985245
ICP_L	0.00722296	0.02598871	0.781660627	0.0048859	0.02601216	0.85526913
ICP_R	0.00953614	0.02522408	0.704991687	0.00635152	0.02523906	0.80122565
IFO_L	0.0213219	0.02186576	0.329031092	-0.02468619	0.0198384	0.21353463
IFO_R	0.05159136	0.02237259	0.021055099	0.0039765	0.02024743	0.84372772
MCP	0.0200022	0.02486488	0.420407828	0.01139166	0.0248281	0.6457187
ML_L	0.01751892	0.02446776	0.47337986	0.01332916	0.02447536	0.58560167
ML_R	0.03977632	0.02350662	0.090469323	0.03542914	0.02351144	0.13169515
PCR_L	0.0827851	0.02112376	**8.87842E-05**	-0.00075445	0.01288297	0.9534157
PCR_R	0.09555443	0.02111827	**6.04238E-06**	0.01081914	0.01260267	0.39068057
PCT	0.06162432	0.02506962	0.013927718	0.04990121	0.02498225	0.04568442
PLIC_L	0.00611375	0.02044753	0.76410479	-0.02425234	0.01953583	0.21471535
PLIC_R	0.00125438	0.02209615	0.953735567	-0.03312851	0.02101018	0.11504552
PTR_L	0.07517115	0.02048301	**0.000241929**	-0.00339655	0.013112	0.7959227
PTR_R	0.07855885	0.02024707	**0.00010409**	0.0015333	0.01312304	0.90639131
RLIC_L	0.0202804	0.021525	0.345515776	-0.0329204	0.01871199	0.07860308
RLIC_R	0.06784148	0.02231575	0.002357324	0.00770708	0.01882604	0.68235725
SCC	0.11112243	0.0195618	**1.35123E-08**	0.03190567	0.01148227	0.00545917
SCP_L	-0.01779885	0.02455409	0.468634225	-0.0214429	0.02456519	0.38265355
SCP_R	-0.0211567	0.02303085	0.358346961	-0.02350579	0.02304622	0.30771415
SCR_L	0.08452362	0.02020327	**2.86868E-05**	0.00682003	0.01285528	0.5954101
SCR_R	0.10071942	0.02065433	**1.08272E-06**	0.01984192	0.01277285	0.12004454
SFO_L	0.04130421	0.01933457	0.032606721	-0.01581396	0.01558293	0.31021862
SFO_R	0.06377439	0.01946202	0.001047908	0.00552441	0.01557517	0.72253138
SLF_L	0.09717597	0.02280858	**2.03319E-05**	0.01521037	0.01595652	0.341136
SLF_R	0.08077503	0.0228385	**0.000402539**	-0.00301484	0.01563544	0.84615535
SS_L	0.05779406	0.02287884	0.011482696	-0.00083948	0.01968239	0.96601491
SS_R	0.06036388	0.02202325	0.006098032	0.00223103	0.01875124	0.905352
TAP_L	0.10272383	0.02222188	**3.77828E-06**	0.02733102	0.01640267	0.09574625
TAP_R	0.09719145	0.02024661	**1.58346E-06**	0.02836662	0.01487573	0.05652573
UNC_L	-0.02647163	0.02553683	0.30171386	-0.04099545	0.02539594	0.10704912
UNC_R	-0.02238699	0.02535852	0.379683142	-0.0320805	0.02530872	0.20599915

Linear mixed models with smoking, age, and cohort as independent variable and WMH volume in 50 ROIs as dependent variable, before and after additional correction for total WMH volume. Uncorrected p-values are provided. To correct for multiple comparisons, a Bonferroni correction for including 50 ROIs was performed. As such, an uncorrected p-value of 0.001 was considered statistically significant. Significant p-values are shown in bold.

Table S4. Linear mixed models for diabetes mellitus

**DM**	Before correction for total WMH volume			After correction for total WMH volume		
	Coefficient	SE	p-value	Coefficient	SE	p-value
MCP	0.0003204	0.02131787	0.988743838	-0.00527802	0.02126518	0.80352793
ML_L	-0.04363872	0.02098366	0.037365295	-0.04695643	0.02097258	0.02500616
ICP_R	0.00819446	0.02158633	0.706001075	0.00673086	0.02159093	0.75707834
ICP_L	-0.0175196	0.02218092	0.420367748	-0.01859272	0.02216811	0.39157935
SCP_R	-0.00979582	0.01970862	0.618317174	-0.01115951	0.0197134	0.57047566
SCP_L	-0.03082366	0.0210206	0.14206842	-0.03268243	0.02102275	0.11960374
CP_R	-0.0233789	0.02026378	0.24877755	-0.02702761	0.02024174	0.18213531
CP_L	-0.01714507	0.01979872	0.386740858	-0.02052675	0.01978084	0.29983849
ALIC_R	0.06888625	0.01651173	**3.04993E-05**	0.03310294	0.0130763	0.0114139
ALIC_L	0.04748729	0.01593843	0.002900096	0.01300229	0.01262509	0.3035631
PLIC_R	0.00743166	0.01898563	0.69685716	-0.01393868	0.01802195	0.43782867
PCT	0.01170815	0.0214905	0.587206462	0.004306	0.02140093	0.84233416
PLIC_L	-0.02217785	0.01758407	0.206691551	-0.0408646	0.01678573	0.01484726
RLIC_R	-0.01934557	0.01916054	0.311557634	-0.05518159	0.01623923	**0.00067538**
RLIC_L	-0.02179527	0.0185539	0.239413369	-0.05441336	0.01611881	**0.0007319**
ACR_R	0.06754542	0.01712932	**8.12235E-05**	0.02173195	0.01122027	0.05299895
ACR_L	0.07265856	0.01730962	**2.72656E-05**	0.02577443	0.01113631	0.02072347
SCR_R	0.07171238	0.01794951	**6.53095E-05**	0.02159043	0.01104861	0.05095456
SCR_L	0.05358716	0.01753928	0.00226093	0.00542365	0.01107605	0.62552262
PCR_R	0.04060273	0.01827839	0.026483456	-0.01101944	0.01079774	0.30771329
PCR_L	0.03912263	0.01827766	0.032441975	-0.01238102	0.01099208	0.25969543
PTR_R	0.0183572	0.01750943	0.295420908	-0.02873756	0.01118523	0.01021031
GCC	0.07845045	0.01541599	**3.65799E-07**	0.0391008	0.01068636	**0.00025491**
PTR_L	0.0193839	0.01766868	0.273296397	-0.02863038	0.01117397	0.01041984
SS_R	-0.01672901	0.01899918	0.377472835	-0.05152508	0.01610951	0.0013898
SS_L	-0.00680416	0.0197401	0.728604213	-0.04220657	0.01688849	0.01247833
EC_R	0.04740948	0.01984392	0.017042446	0.00885069	0.01662305	0.59708057
EC_L	0.03963651	0.01996989	0.047525187	-0.00031512	0.01649323	0.98229714
CGC_R	0.00488448	0.02163185	0.823886393	-0.00950322	0.02115748	0.65399786
CGC_L	0.01811006	0.02155854	0.40134013	0.00344651	0.02100706	0.86396047
CGH_R	-0.01226121	0.02090494	0.557303552	-0.01826608	0.02083577	0.38101815
CGH_L	0.01175936	0.02098621	0.575448391	0.00622947	0.02092698	0.76538139
FXST_R	0.00433465	0.02125005	0.83945892	-0.00766661	0.02094687	0.71503774
BCC	0.08179431	0.01770141	**3.88274E-06**	0.03245686	0.01083234	0.00274154
FXST_L	-0.01214527	0.02093266	0.560963154	-0.02382794	0.02065073	0.24874994
SLF_R	0.05380106	0.01961328	0.006157625	0.00478355	0.01350755	0.72146123
SLF_L	0.03083687	0.0196007	0.116335237	-0.01769948	0.01372269	0.1975155
SFO_R	0.06509726	0.01686338	**0.000114208**	0.0287156	0.01338094	0.03198798
SFO_L	0.04100815	0.0167116	0.014178582	0.00547942	0.01337274	0.68282279
IFO_R	0.06079887	0.01922178	0.001572243	0.03081188	0.01728434	0.07501607
IFO_L	0.05802668	0.01877351	0.002007264	0.02902547	0.01691162	0.08641751
UNC_R	0.03666006	0.02168419	0.091862128	0.03101955	0.02162507	0.15236896
UNC_L	0.00604666	0.02184212	0.785672917	-0.00219058	0.02171936	0.91660862
TAP_R	-0.02090897	0.01751549	0.231972196	-0.06299995	0.0127222	**7.4477E-07**
SCC	0.05134847	0.01692957	0.002440664	0.00307339	0.00980009	0.7529389
TAP_L	0.00755885	0.01910077	0.692131328	-0.03821084	0.01398895	0.00634976
FX	-0.00449966	0.01935823	0.815135303	-0.01306673	0.01921198	0.49569792
CST_R	0.00995159	0.02183981	0.650646276	0.00309225	0.0217662	0.88964645
CST_L	0.04411714	0.02178621	0.043101985	0.03835913	0.02173732	0.07806957
ML_R	-0.00828688	0.02018008	0.680037488	-0.01157935	0.02016807	0.56453444

Linear mixed models with diabetes mellitus, age, and cohort as independent variable and WMH volume in 50 ROIs as dependent variable, before and after additional correction for total WMH volume. Uncorrected p-values are provided. To correct for multiple comparisons, a Bonferroni correction for including 50 ROIs was performed. As such, an uncorrected p-value of 0.001 was considered statistically significant. Significant p-values are shown in bold.

Table S5. Linear mixed models for hypercholesterolemia

**HC**	Before correction for total WMH volume			After correction for total WMH volume		
	Coefficient	SE	p-value	Coefficient	SE	p-value
ACR_L	0.02198982	0.01502397	0.143194981	0.01481893	0.00966813	0.12516091
ACR_R	0.00130903	0.01485741	0.929207302	-0.00552817	0.00972025	0.57118711
ALIC_L	-0.00579191	0.01384412	0.675575226	-0.01110566	0.01095322	0.31045607
ALIC_R	-0.00696994	0.01433388	0.62668736	-0.01247723	0.01134665	0.2713121
BCC	-0.00313328	0.01535934	0.837647928	-0.01091474	0.00938969	0.24396681
CGC_L	0.02510451	0.01863727	0.175581522	0.02224786	0.01814878	0.21703112
CGC_R	0.0108114	0.0187283	0.560109514	0.00949792	0.01826932	0.59696365
CGH_L	0.00093724	0.01818243	0.957061773	4.3035E-05	0.01812128	0.99628098
CGH_R	-0.00742672	0.01811775	0.681679477	-0.00843402	0.01805071	0.63995216
CP_L	-0.01735811	0.01714829	0.312919624	-0.01789188	0.0171254	0.29757857
CP_R	-0.00069216	0.0175604	0.971311109	-0.00125126	0.017534	0.94591148
CST_L	0.00208379	0.01889904	0.912759958	0.00114557	0.01884567	0.95225617
CST_R	-0.01741693	0.01894441	0.358034213	-0.01843824	0.0188713	0.32867811
EC_L	0.00599384	0.01733173	0.729612897	-0.00078799	0.01425537	0.95353448
EC_R	0.00934302	0.01721483	0.586984886	0.00377591	0.01435396	0.79065716
FX	-0.01505456	0.01679423	0.368231612	-0.01642724	0.0166638	0.32243992
FXST_L	0.00966105	0.01814094	0.591694731	0.00795073	0.01788367	0.65346401
FXST_R	0.03031486	0.01841053	0.098465671	0.02847017	0.01813889	0.11513505
GCC	0.02328682	0.01338407	0.081855841	0.01723595	0.00926678	0.06286846
ICP_L	0.02167926	0.01880934	0.243404594	0.02144766	0.01874674	0.24645297
ICP_R	0.02147016	0.01869682	0.250502853	0.02119927	0.01869202	0.25640509
IFO_L	-0.00831776	0.01629751	0.609964189	-0.01272047	0.01466823	0.38605492
IFO_R	0.01252921	0.01668087	0.452553421	0.0079228	0.01499173	0.5972123
MCP	-0.01014777	0.01847978	0.586147506	-0.01094163	0.01842575	0.55599814
ML_L	-0.01858686	0.01819838	0.306779648	-0.01910483	0.01818152	0.29299462
ML_R	0.00151419	0.01748344	0.931176707	0.00101674	0.0174668	0.95381881
PCR_L	0.0036059	0.01584589	0.820178779	-0.00448075	0.0095246	0.63686431
PCR_R	0.00407569	0.01584332	0.79654307	-0.0036443	0.00935689	0.69896642
PCT	0.00302375	0.01863532	0.871526032	0.00184618	0.01854698	0.92127289
PLIC_L	-0.02789329	0.01525722	0.067499839	-0.03076408	0.01455923	0.03457504
PLIC_R	-0.02996986	0.01647859	0.06897425	-0.03318941	0.01563696	0.0338124
PTR_L	-0.01453593	0.01532554	0.342748049	-0.02209342	0.00968887	0.02251118
PTR_R	-0.00681335	0.0151761	0.654519607	-0.01346382	0.00969096	0.16704245
RLIC_L	-0.01051953	0.01609383	0.513285832	-0.01555822	0.01397071	0.26527042
RLIC_R	-0.0292661	0.01662468	0.078558696	-0.03442994	0.01408008	0.01461159
SCC	-0.0076359	0.01468306	0.602739537	-0.0151812	0.0084962	0.07376342
SCP_L	-0.01205735	0.01822345	0.508657198	-0.01235699	0.01821775	0.4980118
SCP_R	-0.00077355	0.01708505	0.96428674	-0.00098322	0.01708253	0.9544905
SCR_L	0.01039664	0.01521825	0.494347359	0.00304339	0.00961206	0.75111386
SCR_R	0.01674829	0.01556811	0.281787803	0.00929019	0.0095692	0.33036918
SFO_L	-0.00737232	0.01450718	0.611122621	-0.01286318	0.01159595	0.26703497
SFO_R	0.00326688	0.01463034	0.823091091	-0.00228466	0.0115975	0.84419014
SLF_L	0.02348775	0.01700166	0.166947641	0.01600314	0.01188377	0.17764788
SLF_R	0.04583877	0.01700525	0.00699595	0.03819305	0.0117072	0.00109671
SS_L	-0.01152185	0.01712108	0.50051019	-0.01739266	0.01463442	0.23373059
SS_R	-0.01326717	0.01647476	0.42136907	-0.01868803	0.01395465	0.1810039
TAP_L	0.00051647	0.01654611	0.975697724	-0.00639611	0.01213437	0.59792333
TAP_R	0.00981066	0.01518332	0.516461881	0.00301049	0.01104694	0.78370326
UNC_L	-0.02914703	0.01891898	0.122113148	-0.03139792	0.01877787	0.09269079
UNC_R	0.00494222	0.01877128	0.799885932	0.00287218	0.01868698	0.88979336

Linear mixed models with hypercholesterolemia, age, and cohort as independent variable and WMH volume in 50 ROIs as dependent variable, before and after additional correction for total WMH volume. Uncorrected p-values are provided. To correct for multiple comparisons, a Bonferroni correction for including 50 ROIs was performed. As such, an uncorrected p-value of 0.001 was considered statistically significant. Significant p-values are shown in bold.

Table S6. Linear mixed models for obesity

**Obesity**	Before correction for total WMH volume			After correction for total WMH volume		
	Coefficient	SE	p-value	Coefficient	SE	p-value
ACR_L	0.03565326	0.01613991	0.027162466	0.0175622	0.01055191	0.09590213
ACR_R	0.03873041	0.01584033	0.014507994	0.02116777	0.01052428	0.04435863
ALIC_L	0.0175888	0.01493802	0.238856394	0.00432533	0.01196417	0.7172773
ALIC_R	0.04064847	0.01533765	0.008043477	0.02691096	0.01222026	0.02762808
BCC	0.03497751	0.01671266	0.036393584	0.0155137	0.01030628	0.13224083
CGC_L	0.0006587	0.01990105	0.974784837	-0.00621583	0.01932756	0.74697645
CGC_R	0.03267726	0.01996517	0.101669459	0.0265196	0.01948519	0.17308
CGH_L	0.00918805	0.01938475	0.635156291	0.00680046	0.01931641	0.72426416
CGH_R	-0.01145484	0.01933347	0.554618688	-0.01388829	0.01926007	0.47209586
CP_L	0.041229	0.01829708	0.024212642	0.03970909	0.01826961	0.02969828
CP_R	0.03354008	0.01872916	0.073276806	0.03193026	0.0186988	0.08762634
CST_L	0.03710957	0.02031169	0.067880488	0.03503114	0.02026527	0.08409791
CST_R	0.01477339	0.02027206	0.4663322	0.0122067	0.02019842	0.54573682
EC_L	0.0499707	0.01854987	0.007061899	0.03541769	0.01541434	0.02135706
EC_R	0.02341424	0.01838979	0.202985925	0.00908694	0.01546097	0.55519938
FX	-0.00763379	0.01787508	0.669964785	-0.0109974	0.01773175	0.53571372
FXST_L	-0.02275117	0.01929386	0.238438261	-0.02734845	0.01903678	0.15099446
FXST_R	-0.04774736	0.01963444	0.015021129	-0.05277399	0.01933073	0.00633871
GCC	0.02198033	0.0142999	0.124372456	0.00678931	0.00998284	0.49677496
ICP_L	0.02322642	0.02052188	0.258853222	0.02292634	0.02051749	0.26516283
ICP_R	0.00970536	0.01996937	0.627857079	0.00908534	0.01996733	0.65005827
IFO_L	0.07465128	0.01745155	**1.8927E-05**	0.06391951	0.01586608	**5.6026E-05**
IFO_R	0.05076115	0.01786775	0.004492608	0.03958167	0.0161681	0.01431139
MCP	0.03729867	0.01964865	0.057707357	0.03534073	0.01960656	0.07151709
ML_L	-0.00524892	0.01945589	0.786662044	-0.00618468	0.019448	0.74977363
ML_R	-0.00754483	0.01857181	0.684052306	-0.00850402	0.01856278	0.646328
PCR_L	-0.01012334	0.01695234	0.550369381	-0.02992924	0.01035747	0.00387013
PCR_R	-0.00180805	0.01694284	0.914192655	-0.02193034	0.01012479	0.03020448
PCT	0.00068815	0.02011694	0.973340988	-0.00204075	0.02003459	0.91821343
PLIC_L	0.02519966	0.01615911	0.118831064	0.01818975	0.01545116	0.23894261
PLIC_R	0.03407073	0.01749806	0.051504772	0.02620943	0.0166718	0.11584024
PTR_L	0.01346844	0.01647041	0.413089849	-0.00518764	0.01051651	0.62346093
PTR_R	-0.00056205	0.01632499	0.971922553	-0.01913435	0.01053619	0.06909383
RLIC_L	-0.01668515	0.0169916	0.326214052	-0.02901432	0.01478837	0.04985497
RLIC_R	0.0206845	0.01776087	0.244359332	0.00665871	0.01503936	0.65833698
SCC	0.00893419	0.01589721	0.574315258	-0.01013717	0.00927515	0.27450674
SCP_L	0.01061076	0.01944469	0.585814492	0.00965425	0.01943633	0.61996592
SCP_R	0.0090644	0.01827313	0.620255348	0.00855953	0.01827205	0.63988033
SCR_L	0.02975929	0.01628273	0.067591973	0.01124923	0.01042057	0.28006798
SCR_R	0.04094394	0.01664899	0.013945192	0.02157076	0.01034388	0.03710772
SFO_L	0.00837183	0.01556472	0.590368013	-0.00528312	0.0125452	0.67410447
SFO_R	0.02263886	0.01555056	0.145464688	0.00873367	0.01240698	0.48148148
SLF_L	0.02293484	0.01821642	0.208056452	0.00406809	0.01283903	0.74995317
SLF_R	0.03004479	0.01817792	0.098537605	0.01071043	0.01252595	0.39235412
SS_L	-0.02384598	0.01816037	0.189382108	-0.03762637	0.01554312	0.01556422
SS_R	-0.02199379	0.0174519	0.207611747	-0.03553251	0.01483152	0.01662444
TAP_L	0.01607929	0.01768031	0.363894402	-0.00137995	0.01301881	0.91548272
TAP_R	-0.00721584	0.01619132	0.65502206	-0.02355376	0.01183739	0.04649057
UNC_L	0.01336089	0.02020726	0.505320096	0.01037469	0.02008767	0.60112737
UNC_R	0.01402565	0.02003759	0.481566177	0.01206599	0.01998155	0.54268713

Linear mixed models with obesity, age, and cohort as independent variable and WMH volume in 50 ROIs as dependent variable, before and after additional correction for total WMH volume. Uncorrected p-values are provided. To correct for multiple comparisons, a Bonferroni correction for including 50 ROIs was performed. As such, an uncorrected p-value of 0.001 was considered statistically significant. Significant p-values are shown in bold.

Table S7. Linear mixed models for history of cardiovascular disease

**CVD**	Before correction for total WMH volume			After correction for total WMH volume		
	Coefficient	SE	p-value	Coefficient	SE	p-value
MCP	-0.02601652	0.03391766	0.444048131	-0.03319934	0.03377027	0.32586172
ML_L	-0.06018	0.03347216	0.073259785	-0.06365758	0.03343703	0.05782428
ICP_R	0.02265069	0.03461928	0.507761341	0.02068315	0.03461694	0.54493252
ICP_L	0.04841691	0.0350071	0.15578322	0.04756493	0.03495531	0.15868742
SCP_R	-0.03163737	0.03154203	0.318073043	-0.03315567	0.03154196	0.29522429
SCP_L	-0.03959926	0.0336709	0.242540462	-0.04190384	0.03366284	0.21583154
CP_R	0.02061527	0.03476122	0.548512389	0.01317484	0.03463684	0.69965506
CP_L	-0.0750239	0.03476093	0.031604177	-0.08290232	0.03461634	0.01690896
ALIC_R	0.0305835	0.02653818	0.248447688	-0.00444174	0.02062098	0.83013622
ALIC_L	0.06692056	0.02486741	0.007097399	0.03464943	0.01951445	0.07565686
PLIC_R	-0.045582	0.02989943	0.128434856	-0.06622019	0.02814092	0.01891943
PCT	-0.07826519	0.03420621	0.02239164	-0.08597667	0.03400795	0.01161935
PLIC_L	0.01248748	0.02863759	0.660307129	-0.00760564	0.02693153	0.78082161
RLIC_R	0.05136393	0.02968107	0.082890736	0.01900363	0.02529165	0.44991951
RLIC_L	0.05860483	0.02918052	0.044294268	0.02896428	0.02543805	0.25330664
ACR_R	0.04143323	0.02715699	0.126595348	-0.00258411	0.01757175	0.88185715
ACR_L	0.05826745	0.02751399	0.034048108	0.01353397	0.01754624	0.44061721
SCR_R	0.05904548	0.02839174	0.037345038	0.01171268	0.01747485	0.50270904
SCR_L	0.06806895	0.02771853	0.013986703	0.02325104	0.0176966	0.18849988
PCR_R	0.09574876	0.02887503	**0.000903571**	0.04490925	0.0170309	0.00847598
PCR_L	0.08981852	0.0289266	0.001888992	0.04086641	0.01753641	0.01982711
PTR_R	0.03569173	0.02798432	0.20068079	-0.01125799	0.01774793	0.52485758
GCC	0.07564741	0.02598599	0.003579573	0.03621341	0.01785665	0.04230622
PTR_L	0.09718964	0.02884201	**0.000746254**	0.04932734	0.01825927	0.00693372
SS_R	0.07368954	0.03164067	0.019604981	0.03884641	0.02715544	0.15152636
SS_L	0.05360485	0.03242789	0.097326853	0.01956513	0.02799179	0.48103701
EC_R	-0.00929733	0.03167909	0.772630152	-0.04391332	0.02661699	0.10080978
EC_L	0.01314827	0.03192747	0.677347934	-0.02329385	0.0263593	0.38149453
CGC_R	0.04947119	0.03467944	0.152500097	0.03320987	0.03398469	0.32908579
CGC_L	0.00756664	0.03496621	0.821131991	-0.01388409	0.034022	0.68557533
CGH_R	0.01287781	0.03333889	0.696685734	0.00508604	0.03316248	0.87613805
CGH_L	0.0118128	0.03423497	0.725103788	0.00436531	0.03408668	0.89359287
FXST_R	-0.01384073	0.03395088	0.686158292	-0.02704685	0.03347219	0.41936833
BCC	0.02716671	0.02840953	0.336815153	-0.01994634	0.01741395	0.25416665
FXST_L	-0.04785848	0.0336082	0.155450416	-0.062525	0.03298759	0.05810958
SLF_R	0.03540902	0.03132577	0.256759158	-0.01550865	0.02163806	0.46871152
SLF_L	0.04343493	0.0312634	0.163659728	-0.00550183	0.02206061	0.79873668
SFO_R	0.01066123	0.02739084	0.6961223	-0.02552793	0.0214131	0.23277945
SFO_L	0.05083544	0.02600739	0.050460953	0.01759096	0.02060642	0.39283623
IFO_R	0.01233547	0.02990729	0.678408446	-0.01544459	0.02680161	0.56561334
IFO_L	0.0363981	0.02906189	0.209767414	0.0092503	0.02602505	0.72138621
UNC_R	-0.0920478	0.03527453	0.009332096	-0.09858857	0.03510997	0.00518704
UNC_L	0.00959293	0.035153	0.780935525	0.00304501	0.03501257	0.92623036
TAP_R	0.02310259	0.02853751	0.415539163	-0.01929583	0.02087083	0.35488144
SCC	0.07773016	0.0276209	0.004816706	0.02904039	0.01595088	0.06831039
TAP_L	0.05699466	0.03155353	0.069742107	0.00819812	0.02323759	0.72567282
FX	-0.06789191	0.02999458	0.024010642	-0.07556326	0.02978054	0.01137014
CST_R	0.00778692	0.03489928	0.819627052	0.00074396	0.03473902	0.9789504
CST_L	-0.04597573	0.03475619	0.187425666	-0.05210781	0.03463538	0.13369413
ML_R	-0.04866214	0.032188	0.13190758	-0.05179534	0.03216033	0.10840566

Linear mixed models with history of cardiovascular disease, age, and cohort as independent variable and WMH volume in 50 ROIs as dependent variable, before and after additional correction for total WMH volume. Uncorrected p-values are provided. To correct for multiple comparisons, a Bonferroni correction for including 50 ROIs was performed. As such, an uncorrected p-value of 0.001 was considered statistically significant. Significant p-values are shown in bold.

Table S8. Linear mixed models for the vascular risk compound score.

**VRCS**	Before correction for total WMH volume			After correction for total WMH volume		
	Coefficient	SE	p-value	Coefficient	SE	p-value
ACR_L	0.06416748	0.00689963	**1.57426E-20**	0.01949229	0.00445759	**1.2349E-05**
ACR_R	0.05681486	0.00683175	**9.76498E-17**	0.01314164	0.00449111	0.00343895
ALIC_L	0.04381524	0.00636838	**6.19421E-12**	0.01091412	0.00505926	0.0310473
ALIC_R	0.04917466	0.00659418	**9.28359E-14**	0.01509026	0.0052396	0.00399582
BCC	0.06141485	0.00705153	**3.34683E-18**	0.0142388	0.004332	0.00101734
CGC_L	0.02355782	0.00859479	0.006055382	0.00753389	0.00839253	0.36503415
CGC_R	0.02222585	0.00862247	0.009911	0.00750541	0.00844968	0.37263874
CGH_L	0.00793798	0.00837015	0.341828227	0.00234196	0.00836223	0.77756778
CGH_R	-0.00097106	0.00833586	0.907655846	-0.00695693	0.00832441	0.40362869
CP_L	-0.00288257	0.00789378	0.717409253	-0.00624561	0.00790164	0.43122927
CP_R	0.00119671	0.00808113	0.879561397	-0.00244309	0.00808778	0.76532413
CST_L	0.01755479	0.00868915	0.043394549	0.01202504	0.00868526	0.16652924
CST_R	0.00518742	0.00871244	0.551701296	-0.00124061	0.00869989	0.88581435
EC_L	0.05871086	0.00795481	**1.65905E-13**	0.02050442	0.00658995	0.00189775
EC_R	0.05646188	0.00790219	**9.39897E-13**	0.01974525	0.00663794	0.00297753
FX	0.0037929	0.00771819	0.623949555	-0.00436321	0.00767571	0.56879661
FXST_L	-0.008479	0.00834584	0.310482086	-0.02004472	0.00824964	0.01521273
FXST_R	0.0040262	0.00847292	0.632927871	-0.00800047	0.0083693	0.34070944
GCC	0.05262489	0.00614465	**1.17931E-17**	0.01517202	0.00427635	**0.00038946**
ICP_L	0.00448985	0.00882125	0.610154283	0.00338155	0.00883126	0.70359257
ICP_R	0.00762361	0.00860432	0.375955048	0.00596723	0.00862186	0.48953056
IFO_L	0.04585062	0.00748826	**9.37084E-10**	0.01845728	0.00677073	0.00641988
IFO_R	0.04312581	0.00766514	**1.87065E-08**	0.01477236	0.00691456	0.0327278
MCP	0.00553406	0.00850063	0.513541136	4.5962E-05	0.00849518	0.99379543
ML_L	-0.01076916	0.0083696	0.197928077	-0.0139612	0.00838074	0.09546356
ML_R	-0.00045753	0.00804896	0.954259884	-0.00359663	0.0080593	0.6546738
PCR_L	0.0433329	0.00728678	**2.78887E-09**	-0.00590177	0.00440529	0.1799441
PCR_R	0.04699388	0.00728899	**1.16938E-10**	-0.00266344	0.00432197	0.53821318
PCT	0.00498695	0.00856849	0.560866293	-0.00211971	0.00854779	0.80326097
PLIC_L	0.01152395	0.00700679	0.100044451	-0.00619833	0.00670355	0.35482226
PLIC_R	0.01327303	0.00756806	0.07948159	-0.00691408	0.00720103	0.33654109
PTR_L	0.03613472	0.00703858	**2.86531E-07**	-0.00988308	0.00447058	0.02712649
PTR_R	0.02884762	0.00698518	**3.63631E-05**	-0.01641876	0.00447268	**0.00024645**
RLIC_L	0.01588873	0.00739033	0.031590018	-0.0151053	0.00643875	0.01891249
RLIC_R	0.02739909	0.00763971	**0.000336261**	-0.0068487	0.00649496	0.29140164
SCC	0.04319324	0.00674952	**1.59754E-10**	-0.00330536	0.00392031	0.39964591
SCP_L	-0.01082105	0.00838184	0.19657995	-0.01274978	0.00839813	0.12881941
SCP_R	-0.00639295	0.00786065	0.415882847	-0.00775776	0.00787725	0.32445522
SCR_L	0.06125773	0.00698812	**2.02746E-18**	0.01543268	0.00443483	**0.00050389**
SCR_R	0.06867238	0.00714857	**8.56238E-22**	0.02100497	0.00441923	**2.0233E-06**
SFO_L	0.04037285	0.00667265	**1.47469E-09**	0.00647811	0.00535332	0.2265372
SFO_R	0.04809539	0.00673019	**9.29168E-13**	0.01341794	0.00535622	0.01227595
SLF_L	0.05876773	0.0078077	**5.47214E-14**	0.01173678	0.00548796	0.03245801
SLF_R	0.06527912	0.00781117	**6.94326E-17**	0.01758797	0.00540388	0.00113163
SS_L	0.01196483	0.00786417	0.128211569	-0.0223644	0.00674678	**0.00091859**
SS_R	0.01097119	0.00756549	0.146865313	-0.02287844	0.00642767	**0.00037482**
TAP_L	0.04218187	0.00761758	**3.08842E-08**	-0.00171756	0.00560098	0.75990429
TAP_R	0.02686759	0.0069922	**0.000121627**	-0.01371832	0.00510096	0.00719006
UNC_L	0.00302562	0.00871295	0.73022032	-0.00504027	0.0086796	0.5583435
UNC_R	0.00850801	0.0086491	0.326762553	0.00284667	0.00864085	0.7456317

Linear mixed models with the vascular risk compound score, age, and cohort as independent variable and WMH volume in 50 ROIs as dependent variable, before and after additional correction for total WMH volume. Uncorrected p-values are provided. To correct for multiple comparisons, a Bonferroni correction for including 50 ROIs was performed. As such, an uncorrected p-value of 0.001 was considered statistically significant. Significant p-values are shown in bold.

Table S9. Association between hypertension and regional WMH volumes, stratified for age, before restandardizing WMH volumes

**ROI**	**Unstratified cohort (n=15532)**	**Age <60 (n=4888)**	**Age 60-80 (n=9796)**	**Age >80 (n=848)**
L external capsule	0.09 (**p<0.001**)	0.04 (**p<0.001**)	0.10 (**p<0.001**) *	0.31 (**p=0.002**)
R external capsule	0.07 (**p<0.001**)	0.06 (**p<0.001**)	0.08 (**p<0.001**) *	0.33 (**p<0.001**)
R superior longitudinal fasciculus	0.04 (**p=0.002**)	0.05 (**p<0.001**)	0.05 (**p<0.001**) *	0.26 (**p<0.001**)
L superior longitudinal fasciculus	0.05 (**p<0.001**)	0.05 (**p<0.001**)	0.05 (**p=0.001**) *	0.15 (**p=0.028**)
L superior corona radiata	0.05 (**p<0.001**)	0.04 (**p<0.001**)	0.06 (**p<0.001**) *	0.03 (p=0.644)
R superior corona radiata	0.04 (**p<0.001**)	0.05 (**p<0.001**)	0.06 (**p<0.001**) *	0.03 (p=0.668)
L anterior corona radiata	0.04 (**p<0.001**)	0.04 (**p=0.001**)	0.05 (**p=0.001**) *	0.07 (p=0.228)
R anterior limb internal capsule	0.05 (**p<0.001**)	0.01 (p=0.286)	0.07 (**p<0.001**) *	0.03 (p=0.744)
L anterior limb internal capsule	0.05 (**p<0.001**)	0.03 (**p=0.017**)	0.05 (**p<0.001**) *	0.08 (p=0.300)
L superior fronto-occipital fasciculus	0.04 (**p<0.001**)	0.04 (**p=0.007**)	0.05 (**p<0.001**) *	0.01 (p=0.900)

Linear mixed models with hypertension as determinant, age, sex, cohort, and total WMH volume as covariate and regional WMH volume as dependent variable, stratified for three age strata. The results of the unstratified analyses are copied from Table 4 and provided as reference. Coefficients and p-values are provided. Note that the z-scores for WMH volumes were not restandardized in this analyses. The results after restandardizing WMH volumes within each age stratum, i.e. creating z-scores with a mean of 0 and an SD of 1, are shown in Table S10. Significant p-values are shown in bold.

Table S10. Association between hypertension and regional WMH volumes, stratified for age, after restandardizing WMH volumes.

**ROI**	**Age <60 (n=4888)**	**Age 60-80 (n=9796)**	**Age >80 (n=848)**
L external capsule	0.10 (**p<0.001**)	0.09 (**p<0.001**)	0.20 (**p=0.002**)
R external capsule	0.15 (**p<0.001**)	0.08 (**p<0.001**)	0.22 (**p<0.001**)
R superior longitudinal fasciculus	0.10 (**p<0.001**)	0.05 (**p<0.001**)	0.20 (**p<0.001**)
L superior longitudinal fasciculus	0.10 (**p<0.001**)	0.05 (**p=0.001**)	0.11 (**p=0.028**)
L superior corona radiata	0.09 (**p<0.001**)	0.06 (**p<0.001**)	0.02 (p=0.644)
R superior corona radiata	0.11 (**p<0.001**)	0.05 (**p<0.001**)	0.02 (p=0.668)
L anterior corona radiata	0.08 (**p=0.001**)	0.05 (**p=0.001**)	0.05 (p=0.228)
R anterior limb internal capsule	0.03 (p=0.286)	0.07 (**p<0.001**)	0.02 (p=0.744)
L anterior limb internal capsule	0.05 (**p=0.017**)	0.05 (**p<0.001**)	0.06 (p=0.300)
L superior fronto-occipital fasciculus	0.07 (**p=0.007**)	0.05 (**p<0.001**)	0.01 (p=0.900)

Linear mixed models with hypertension as determinant, age, sex, cohort, and total WMH volume as covariate and regional WMH volume as dependent variable, stratified for three age strata. In this analysis, the z-scores for regional WMH volumes have been restandardized within each age stratum, meaning that the mean of the dependent variable was 0 and the SD was 1 for each analysis. Significant p-values are shown in bold.

Figure S3. Association between hypertension and regional WMH volumes, stratified for age.

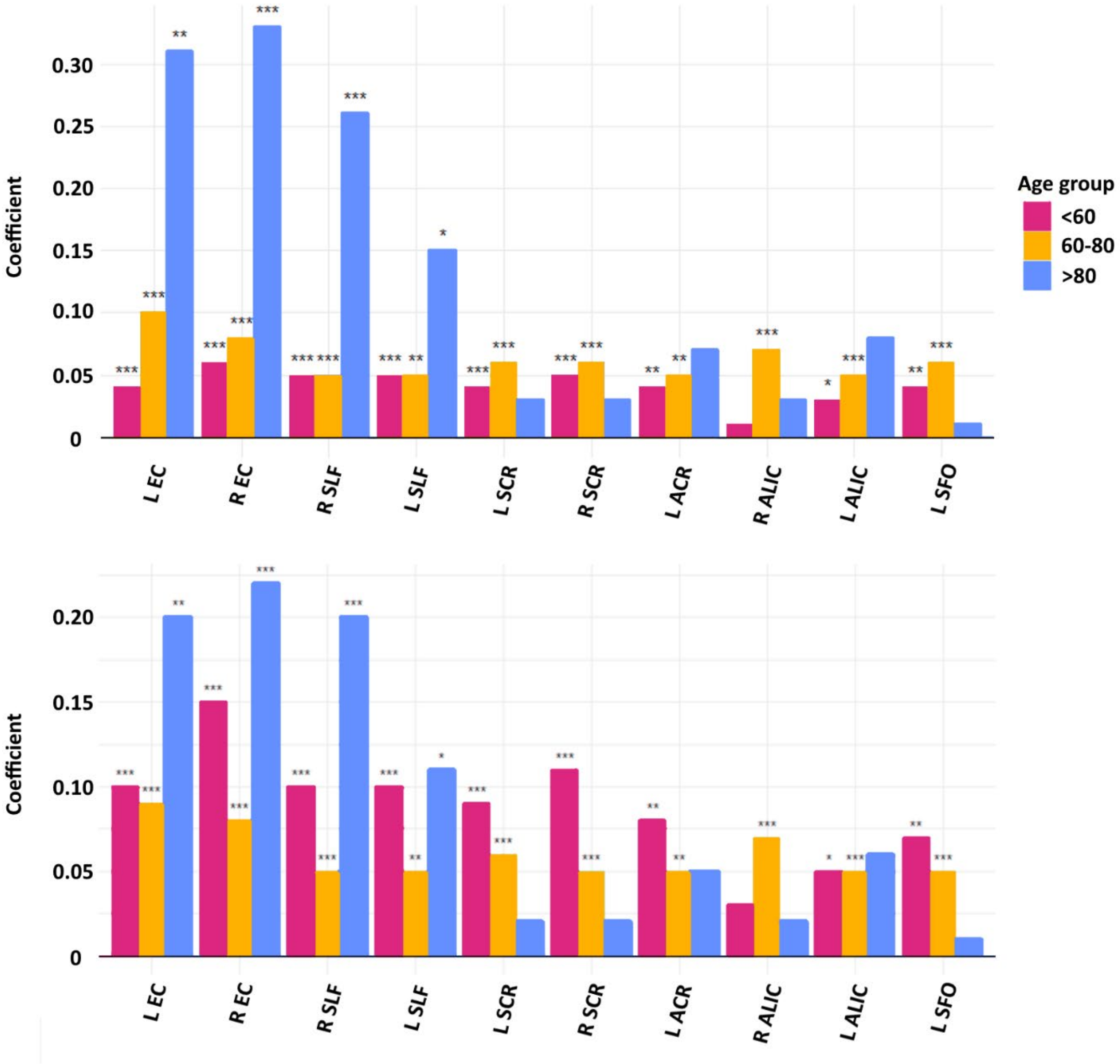

*This figure provides a visual illustration of Tables S9 and S10 for ease of interpretation and comparison of the results before (upper graph) and after (lower graph) restandardizing WMH volumes within age strata. EC: external capsule. SLF: superior longitudinal fasciculus. SCR: superior corona radiata. ACR: anterior corona radiata. ALIC: anterior limb of internal capsule. SFO: superior fronto-occipital fasciculus. * p<0.05, ** p<0.01, *** p<0.001.*

Table S11. Sensitivity analyses: associations between vascular risk factors and regional WMH volumes after excluding patients with a history of prior stroke.

**ROI**	**Hypertension**	**Obesity**	**Smoking**	**Diabetes**
L external capsule	0.08 (**p<0.001**)	-	-	-
R external capsule	0.08 (**p<0.001**)	-	-	-
R superior longitudinal fasciculus	0.06 (**p<0.001**)	-	-	-
L superior longitudinal fasciculus	0.05 (**p<0.001**)	-	-	-
L superior corona radiata	0.04 (**p<0.001**)	-	-	-
R superior corona radiata	0.04 (**p<0.001**)	-	-	-
L anterior corona radiata	0.04 (**p<0.001**)	-	-	-
R anterior limb internal capsule	0.04 (**p<0.001**)	-	-	-
L anterior limb internal capsule	0.04 (**p<0.001**)	-	-	-
L superior fronto-occipital fasciculus	0.04 (**p<0.001**)	-	-	-
L inferior fronto-occipital fasciculus	-	0.06 (**p<0.001**)	-	-
body corpus callosum	-	-	0.05 (**p<0.001**)	-
genu corpus callosum	-	-	-	0.04 (**p<0.001**)

Linear mixed models with vascular risk factors as independent variable, age, sex, cohort, and total WMH volume as covariates and regional WMH volume as dependent variable. All significant main results after correction for total WMH volume (i.e. results shown after the slash in Table 4 of the main text) are included in this sensitivity analyses, in which patients with a history of clinical stroke (n=271, 1.7%) or missing data on prior stroke (n=32) were excluded. In this sensitivity analyses, the results were essentially unchanged. Significant p-values are shown in bold.

Sources of funding for individual cohorts
The Austrian Stroke Prevention Study received support from the Austrian Science Fund (grant numbers P20545, P13180, KLI523, P30134 and I2889-B31). The AUCD was supported by P30 AG072972. The Framingham Heart Study is supported by Contract No. 75N92019D00031 from the National Heart, Lung, and Blood Institute (NHLBI) with additional support from other sources. This manuscript has been reviewed by the Framingham Heart Study for scientific content and consistency of data interpretation with previous Framingham Heart Study publications. The Epidemiology of Dementia in Singapore Study is supported by the National Medical Research Council (NMRC), Singapore (NMRC/CG/NUHS/2010 [grant R-184-006-184-511]).The Hamburg City Health Study is supported by Amgen, Astra Zeneca, Bayer, BASF, Deutsche Gesetzliche Unfallversicherung (DGUV), Deutsches Institut für Ernährungsforschung, the Innovative medicine initiative (IMI) under grant number No. 116074, the Fondation Leducq under grant number 16 CVD 03., the euCanSHare grant agreement under grant number 825903-euCanSHare H2020, Novartis, Pfizer, Schiller, Siemens, Unilever and "Förderverein zur Förderung der HCHS e.V.". The Sydney Memory and Ageing Study (MAS) has been funded by three National Health & Medical Research Council (NHMRC) Program Grants (ID No. ID350833, ID568969, and APP1093083). MRI scans in MAS were processed with the support of NHMRC Project Grants (510175 and 1025243) and an ARC Discovery Project Grant (DP0774213) and John Holden Family Foundation. We also thank the MRI Facility at NeuRA, Sydney. The OATS study has been funded by a National Health & Medical Research Council (NHMRC) and Australian Research Council (ARC) Strategic Award Grant of the Ageing Well, Ageing Productively Program (ID No. 401162); NHMRC Project (seed) Grants (ID No. 1024224 and 1025243); NHMRC Project Grants (ID No. 1045325 and 1085606); and NHMRC Program Grants (ID No. 568969 and 1093083). The LBC1921 was supported by the UK's Biotechnology and Biological Sciences Research Council (BBSRC), The Royal Society, and The Chief Scientist Office of the Scottish Government. The LBC1936 was jointly supported by the BBSRC and the Economic and Social Research Council [BB/W008793/1. to SRC, which supports JMW, MEB, SMM], Age UK (Disconnected Mind project), the Milton Damerel Trust, the Medical Research Council [MRC; G0701120, G1001245, MR/M013111/1, MR/R024065/1], and the University of Edinburgh. The Rotterdam Study is funded by Erasmus Medical Center and Erasmus University, Rotterdam, the Netherlands Organization for the Health Research and Development (ZonMw), the Research Institute for Diseases in the Elderly (RIDE), the Ministry of Education, Culture and Science, the Ministry for Health, Welfare and Sports, the European Commission (DG XII), and the Municipality of Rotterdam. The SABRE study has been supported by the British Heart Foundation, Wellcome Trust, UK Medical Research Council and Diabetes UK. The CNS was supported by the Canadian Institutes of Health Research, the Seaman Family MR Research Centre and the Hopewell Professorship.
