# Supplementary material for: Signature White Matter Hyperintensity Locations Associated With Vascular Risk Factors Derived From 15 653 Individuals
Source: Stroke. 2025 Aug 15;56(10):3047–59. doi: 10.1161/STROKEAHA.125.051159 (PMC12447828; doi:10.1161/STROKEAHA.125.051159)
Supplement: Supplementary file 2 [file str-56-3047-s002.pdf]

STROBE Statement—Checklist of items that should be included in reports of ***cross-sectional studies***

	Item No	Recommendation
**Title and abstract**	1	(*a*) Indicate the study's design with a commonly used term in the title or the abstract *Included in title and abstract*
		(*b*) Provide in the abstract an informative and balanced summary of what was done and what was found *Included in abstract*
**Introduction**		
Background/rationale	2	Explain the scientific background and rationale for the investigation being reported *Included in introduction (page 4 of manuscript)*
Objectives	3	State specific objectives, including any prespecified hypotheses *Included in introduction (page 4 of manuscript)*
**Methods**		
Study design	4	Present key elements of study design early in the paper *Methods (page 5 of manuscript)*
Setting	5	Describe the setting, locations, and relevant dates, including periods of recruitment, exposure, follow-up, and data collection *Table S1*
Participants	6	(*a*) Give the eligibility criteria, and the sources and methods of selection of participants *Table S1*
Variables	7	Clearly define all outcomes, exposures, predictors, potential confounders, and effect modifiers. Give diagnostic criteria, if applicable *Methods (page 6 of manuscript)*
Data sources/ measurement	8*	For each variable of interest, give sources of data and details of methods of assessment (measurement). Describe comparability of assessment methods if there is more than one group *Online supplements*
Bias	9	Describe any efforts to address potential sources of bias *Methods (pages 6-7), discussion (pages 14 and 15)*
Study size	10	Explain how the study size was arrived at *Flow chart, figure S1*
Quantitative variables	11	Explain how quantitative variables were handled in the analyses. If applicable, describe which groupings were chosen and why *Methods, section statistics, pages 6 and 7*
Statistical methods	12	(*a*) Describe all statistical methods, including those used to control for confounding *Methods, section statistics, pages 6 and 7*
		(*b*) Describe any methods used to examine subgroups and interactions *Methods, section statistics, pages 6 and 7*
		(*c*) Explain how missing data were addressed *Methods, page 6 and 7*
		(*d*) If applicable, describe analytical methods taking account of sampling strategy *N/A*
		(*e*) Describe any sensitivity analyses *Page 7 of manuscript*

**Results**		
Participants	13*	(a) Report numbers of individuals at each stage of study—eg numbers potentially eligible, examined for eligibility, confirmed eligible, included in the study, completing follow-up, and analysed<br>*Figure S1 and page 5 of manuscript*
		(b) Give reasons for non-participation at each stage<br>*Figure S1 and page 5 of manuscript*
		(c) Consider use of a flow diagram<br>*Figure S1*
Descriptive data	14*	(a) Give characteristics of study participants (eg demographic, clinical, social) and information on exposures and potential confounders<br>*Results, page 9, and Table 2*
		(b) Indicate number of participants with missing data for each variable of interest<br>*Table 2*
Outcome data	15*	Report numbers of outcome events or summary measures<br>*WMH volumes are the outcome of the study, a summary measure is provided in Table 2 and Figure S2*
Main results	16	(*a*) Give unadjusted estimates and, if applicable, confounder-adjusted estimates and their precision (eg, 95% confidence interval). Make clear which confounders were adjusted for and why they were included<br>*Results, Tables 3-6, Tables S2-S11*
		(*b*) Report category boundaries when continuous variables were categorized<br>*Methods and Supplemental methods*
		(*c*) If relevant, consider translating estimates of relative risk into absolute risk for a meaningful time period<br>*N/A*
Other analyses	17	Report other analyses done—eg analyses of subgroups and interactions, and sensitivity analyses<br>*Results, Tables S9 and S10, and S11*
**Discussion**		
Key results	18	Summarise key results with reference to study objectives<br>*Discussion, page 12*
Limitations	19	Discuss limitations of the study, taking into account sources of potential bias or imprecision. Discuss both direction and magnitude of any potential bias<br>*Discussion, pages 15 and 16*
Interpretation	20	Give a cautious overall interpretation of results considering objectives, limitations, multiplicity of analyses, results from similar studies, and other relevant evidence<br>*Discussion*
Generalisability	21	Discuss the generalisability (external validity) of the study results<br>*Discussion, page 15*
**Other information**		
Funding	22	Give the source of funding and the role of the funders for the present study and, if applicable, for the original study on which the present article is based<br>*Pages 17 and 18 and online supplements*

*Give information separately for exposed and unexposed groups.

Note: An Explanation and Elaboration article discusses each checklist item and gives methodological background and published examples of transparent reporting. The STROBE checklist is best used in conjunction with this article (freely available on the Web sites of PLoS Medicine at http://www.plosmedicine.org/, Annals of Internal Medicine at http://www.annals.org/, and Epidemiology at http://www.epidem.com/). Information on the STROBE Initiative is available at www.strobe-statement.org.
